# A review on the mechanisms underlying the antitumor effects of natural products by targeting the endoplasmic reticulum stress apoptosis pathway

**DOI:** 10.3389/fphar.2023.1293130

**Published:** 2023-11-17

**Authors:** Jie-Xiang Zhang, Wei-Chen Yuan, Cheng-Gang Li, Hai-Yan Zhang, Shu-Yan Han, Xiao-Hong Li

**Affiliations:** ^1^ The First Clinical College of Shandong University of Traditional Chinese Medicine, Jinan, China; ^2^ Jiangsu Collaborative Innovation Center of Traditional Chinese Medicine in Prevention and Treatment of Tumor, The First Clinical College of Nanjing University of Chinese Medicine, Nanjing, China; ^3^ The College of Traditional Chinese Medicine, Shandong University of Traditional Chinese Medicine, Jinan, China; ^4^ Shandong University of Traditional Chinese Medicine, Jinan, China; ^5^ Key Laboratory of Carcinogenesis and Translational Research (Ministry of Education), Department of Integration of Chinese and Western Medicine, Peking University Cancer Hospital and Institute, Beijing, China

**Keywords:** endoplasmic reticulum stress, natural products, antitumor, apoptosis, review

## Abstract

Cancer poses a substantial risk to human life and wellbeing as a result of its elevated incidence and fatality rates. Endoplasmic reticulum stress (ERS) is an important pathway that regulates cellular homeostasis. When ERS is under- or overexpressed, it activates the protein kinase R (PKR)-like endoplasmic reticulum kinase (PERK)-, inositol-requiring enzyme 1 (IRE1)- and activating transcription Factor 6 (ATF6)-related apoptotic pathways to induce apoptosis. Tumor cells and microenvironment are susceptible to ERS, making the modulation of ERS a potential therapeutic approach for treating tumors. The use of natural products to treat tumors has substantially progressed, with various extracts demonstrating antitumor effects. Nevertheless, there are few reports on the effectiveness of natural products in inducing apoptosis by specifically targeting and regulating the ERS pathway. Further investigation and elaboration of its mechanism of action are still needed. This paper examines the antitumor mechanism of action by which natural products exert antitumor effects from the perspective of ERS regulation to provide a theoretical basis and new research directions for tumor therapy.

## 1 Introduction

Cancer is a large threat to human health, resulting in 19.29 million cancer diagnoses and 9.96 million fatalities across the globe in 2020, with 49.3% of new cases and 58.3% of deaths occurring in Asia, among the highest in the world; in addition, 28.4 million new cancer cases are expected to occur by 2040, a 47% increase from 2020 (Sung et al., 2021). According to a single estimate, China is approximately 4,820,000 individuals diagnosed with cancer and 3,210,000 fatalities due to cancer in 2022 (Xia C. et al., 2022). In addition, according to the U.S. Cancer Database, as of 1 January 2022, there are more than 18 million Americans with a history of cancer (8.3 million males and 9.7 million females) of which the most common cancer for males is prostate cancer (3,523,230) and for females is breast cancer (4,055,770) (Miller et al., 2022). Hence, tackling this problem and decreasing the occurrence and death rates related to cancer is crucial. The mechanisms and development of cancer are not completely understood. These factors could include smoking, drinking alcohol, genetic predisposition, dietary habits, and nutritional intake. Early cancer detection is rare, and there are limited radical treatment options. Contemporary cancer treatments primarily consist of surgical procedures, radiation therapy, chemotherapy, targeted therapy, and immunotherapy. However, these methods may be accompanied by distinct toxic side effects and adverse reactions, leading to less-than-anticipated outcomes. Therefore, the ability to effectively treat or even eradicate cancer and reduce the accompanying toxic side effects has been a pressing issue. To this end, we have tried various approaches and found that natural products seem to be an effective way.

Natural products are compounds or substances found in living organisms in nature. Natural products are widespread around the world for their suitable antitumor properties, and more than 100 products have demonstrated ideal anticancer effects, mostly from plants and to a lesser extent from ore-based drugs, animals and microorganisms (Fabiani, 2020; Sauter, 2020; Zhou et al., 2022). Natural products have the advantages of stable efficacy, multitargeting and multiple routes of action, as well as a high safety profile due to less irritation to the gastrointestinal tract and less damage to liver and kidney function. Studies have found that many natural products, such as dihydroartemisinin, celastrol, and resveratrol, possess the ability to hinder the spread and growth of tumor cells, promote cancer cell death, improve cancer cell metabolism and regulate oncogenes (Luo et al., 2019; Xiang et al., 2019; Wang et al., 2021). Turmeric extract β-elemene inhibits cancer cell proliferation and promotes apoptosis by regulating vascular endothelial growth factor (VEGF), matrix metalloproteinase, E-cadherin, N-cadherin and vimentin, which are factors related to tumor angiogenesis and metastasis, while enhancing the sensitivity of tumor cells to radiotherapy (Zhai et al., 2019). Amygdalin, which is widely found in the seed kernels of apricot, peach, plum and prune plants, has been demonstrated to trigger the internal mitochondrial apoptotic pathway in HepG2 cells, a type of liver cancer cell, and block cells in the G2/M period to induce apoptosis (El-Desouky et al., 2020). A clinical trial concerning 75 patients with advanced stomach cancer shows that after two cycles of treatment with Javanica oil emulsion injection combined with chemotherapy, the total effective rate of treatment was 85.3%, which indicates its good anti-tumor effect (Liu et al., 2013). Although long-term experiments have been conducted on the antitumor mechanism of action of many natural products, their specific mechanisms of action remain unclear, necessitating additional research.

The endoplasmic reticulum stress (ERS)-induced apoptosis pathway has recently gained increasing recognition in the field of tumor therapy. The endoplasmic reticulum is an essential organelle for synthesizing, folding and modifying proteins in the cell. It is also involved in intracellular material transportation, lipid synthesis and metabolism, and storing Ca^2+^. Under nonstress conditions, three endoplasmic reticulum proteins, protein kinase R (PKR)-like endoplasmic reticulum kinase (PERK), inositol-requiring enzyme 1 (IRE1) and activating transcription Factor 6 (ATF6), specifically bind to the regulatory protein heavy-chain binding protein/glucose-regulated protein 78 (Bip/GRP78), forming a homeostatic complex and remaining in an inactive state. However, if the cell is altered internally as a result of changes in its environment, the aggregation of unfolded and misfolded proteins and Ca^2+^ homeostasis disruption activate the protective ERS pathway. As a result, PERK, IRE1, and ATF6 separate from the GRP78 protein and activate the unfolded protein response (UPR). The UPR helps return misfolded or unfolded proteins to normal, relieving the endoplasmic reticulum load to maintain cellular homeostasis (Oakes and Papa, 2015; Marciniak et al., 2022). However, under- or overactivation of ERS can induce cell death (Walter and Ron, 2011). Tumor cells substantially differ from normal cells and are often exposed to an ischaemic, hypoxic and nutrient-deficient tumor microenvironment (Chen and Cubillos-Ruiz, 2021; Groenendyk et al., 2021), predisposing them to ERS (Oakes, 2020). Thus, regulating the ERS of tumor cells may be a tumor treatment strategy (Kim and Kim, 2018). There is also growing evidence that the modulation of endoplasmic reticulum pressure sensors or UPR-related factors significantly increases the sensitivity of aggressive tumors to cytotoxic drugs, targeted therapies and immunotherapy (Chen and Cubillos-Ruiz, 2021). The effectiveness of standard chemotherapy and cancer immunotherapy can be improved by implementing targeted ERS responses (Cubillos-Ruiz et al., 2017). However, the mechanism of action of natural products that regulate ERS-related apoptotic pathways still needs to be clarified and requires further integration of various reports and pieces of information. Previous studies have described that natural products and traditional Chinese medicines inhibit tumor progression through ERS (Kim and Kim, 2018; Xia F. et al., 2022), but they involve fewer cancer types and fail to summarize the antitumor mechanism of the ERS-meditated apoptosis pathway fully. In this paper, we searched PubMed to collect as much recent literature as possible on the antitumor effects by which natural products exert through the modulation of ERS apoptotic pathway to describe better the mechanism of ERS-related effects and their relationship with antitumor therapy to provide new research ideas for tumor treatment.

## 2 The mechanism of apoptosis induced by ERS

**FIGURE 1:**
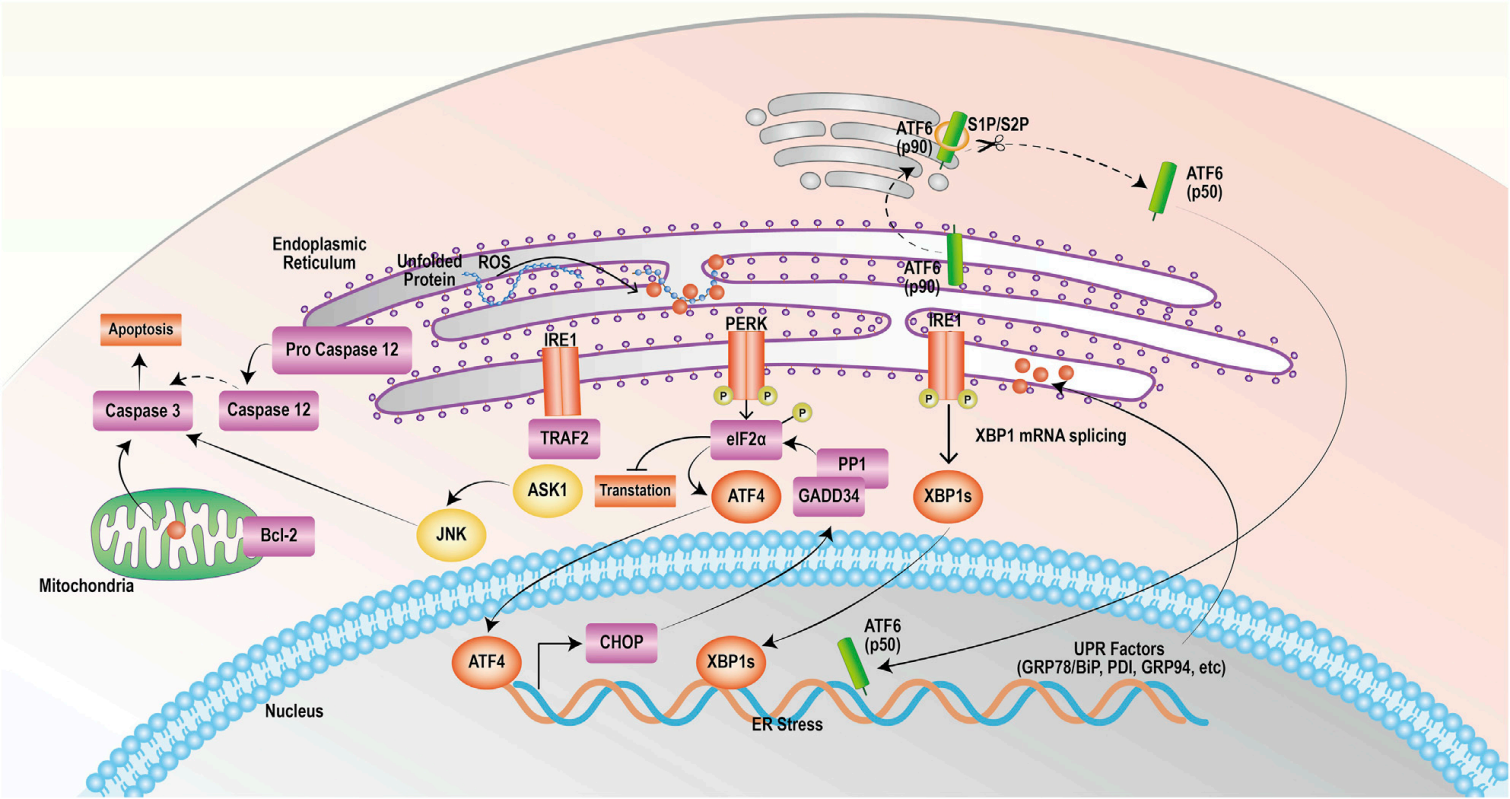
Endoplasmic reticulum stress exerts a mechanism that promotes apoptosis. The onset of the unfolded protein response activates endoplasmic reticulum stress, which mediates the PERK, IRE1, and ATF6 pathways that all induce apoptosis.

PERK, IRE1 and ATF6 bind to the GRP78 protein in a nonactivated state under physiological conditions (Zhao and Ackerman, 2006). ERS triggers the release of PERK, which then phosphorylates its catalytic substrate eukaryotic translation initiation Factor 2 α (eIF2α) through autophosphorylation. Phosphorylated eIF2α can quickly hinder protein synthesis and decrease the burden on the endoplasmic reticulum. Nevertheless, as the stress reaction escalates, excessively stimulated eIF2α stimulates the production of initiating transcription Factor 4 (ATF4), leading to the upregulated expression of the apoptotic signaling molecule C/EBP homologous protein (CHOP)/growth arrest and DNA damage-inducible 153 (GADD153). CHOP can trigger oxidative damage by activating growth arrest and DNA damage protein 34 (GADD34) or suppressing the level of the antiapoptotic protein B-cell lymphoma-2 (Bcl-2), thereby facilitating apoptosis (Boyce and Yuan, 2006; Bobrovnikova-Marjon et al., 2010; Rozpedek et al., 2016). Activation of IRE1 induces its phosphorylation and the formation of dimeric IRE1α, which leads to apoptosis through three pathways. First, IRE1α exhibits nucleic acid endonuclease activity, causing cleavage and maturation of X-box binding protein 1 (XBP1) mRNA and upregulating CHOP transcriptional expression, thereby promoting apoptosis (Li et al., 2019; Jaud et al., 2020). Second, IRE1 interacts with TNF receptor-associated Factor 2 (TRAF2) and recruits apoptosis signal-regulating kinase 1 (ASK1), composing the IRE1-TRAF2-ASK1 complex and activating c-Jun N-terminal kinase (JNK) to induce the JNK apoptotic pathway (Urano et al., 2000a; Urano et al., 2000b). Finally, IRE1 activates caspase-12 by binding to TRAF2, initiating the caspase cascade reaction and inducing apoptosis. ATF6 is dissociated from the GRP78 protein and transferred to the Golgi apparatus, which becomes active through the cleavage of Golgi proteases S1P and S2P; activated ATF6 promotes CHOP transcription and expression, which inhibits Bcl-2 and leads to apoptosis (Wang et al., 2000; Chen et al., 2022). The mechanism of action of targeting ERS to promote apoptosis in tumor cells is shown in Figure 1.

## 3 Application of natural products in inducing apoptosis in tumor cells

**FIGURE 2:**
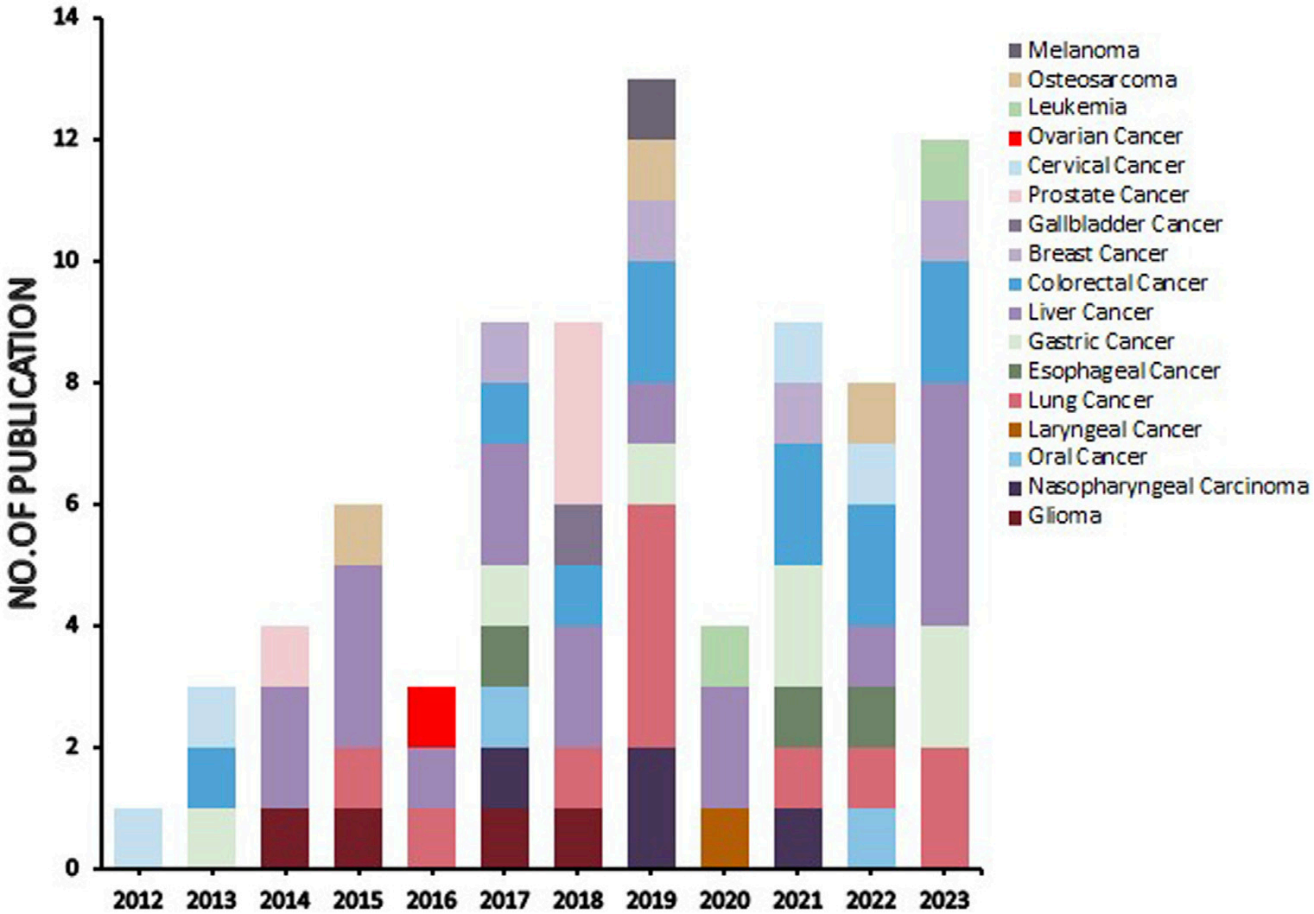
Literature on studies related to natural products targeting endoplasmic reticulum stress for antitumor effects (stacked diagram). The period is set from 1 January 2012 to 20 July 2023. As can be seen from the graph, there is an upward trend in its research heat.

Natural products can target ERS to exert apoptotic effects on tumor cells and have inhibitory effects on various cancers listed below. Figure 2 is demonstrated. A total of 69 natural products promoting apoptosis in tumor cells through the ERS pathway were collected, of which 51 could be searched for structural formulae from Pubchem. The mechanisms of action and structural formulae of the natural products are shown in Table 1.

**TABLE 1:** Mechanism of action of natural products targeting ERS to promote apoptosis in tumor cells and structural formulae of some natural products.

Natural products	Source	Types of natural products	Types of cancer	Cellular/animal models	Mechanism of action of ERS	References
Fatsioside A	Fatsia japonica	Triterpene glycoside	Glioma	U87MG cells	PERK, eIF2α, CHOP↑, cleavage of caspase-4	Pan et al. (2015)
Sinomenine Hydrochloride 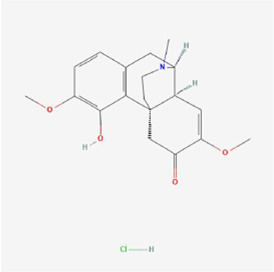	Sinomenium acutum	Morphinans	Glioblastoma	U87 and SF767 cells	Ca2+, GRP78, PERK, IRE1 and CHOP↑	Jiang et al. (2018)
Bufalin 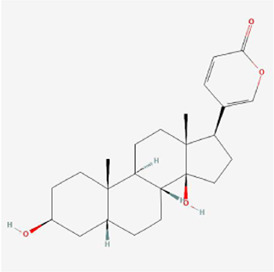	Bufonis Venenum	Glycosides	Glioma	U87MG and LN229 cells	CHOP, ATF6, GRP78 ↑, p-PERK and p-eIF2α↑, cleavage of caspase-4	Shen et al. (2014)
Isochaihulactone (K8) 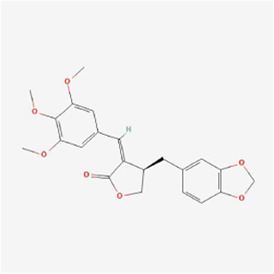	Bupleurum scorzonerifolium Willd.	Benzodioxoles	Glioblastoma multiform	GBM cells /xenograft mice	DDIT3, nag1↑	Tsai et al. (2017b)
Guangsangon E	Leaves of Morus alba L	Diels-Alder adduct	Nasopharyngeal Cancer	CNE1 cells	GRP78, IRE1α, ATF4 and ROS↑	Shu et al. (2021)
Gambogenic acid 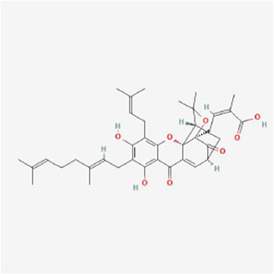	Gamboge	Flavonoid	Nasopharyngeal Cancer	CNE-2Z cells	CHOP, ATF4↑	Su et al. (2019)
PP-22	P.polyphylla var. yunnanensis	Monomers	Nasopharyngeal Cancer	CNE-2 cells	PERK↑, phosphorylation and upregulation of CHOP, BIP, PDI, EROL-LA and IRE-LA	Tan et al. (2019)
Tetrandrine 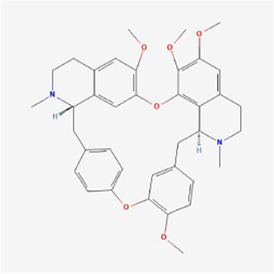	Stephania tetrandra	Alkaloids	Nasopharyngeal Cancer	NPC-TW 039 cells	ROS, Ca2+, GADD153 and GRP78↑	Liu et al. (2017a)
Glaucocalyxin A 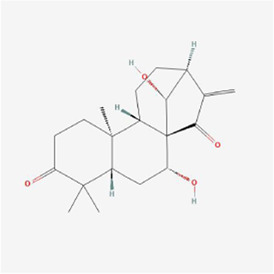	Rabdosia japonica	Terpenoids	Human oral squamous cell cancer	SCC25 and CAL27 cells	PERK-ATF4-CHOP pathway, CHAC1, ROS↑	Wang et al. (2022)
Arsenic-Trioxide (As2O3) 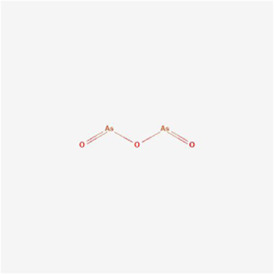	Arsenic-Trioxide	Arsenicals	Oral cancer	Oral cancer cells	GRP78, calpain 1/2 and caspase-3/9/12↑	Tsai et al. (2017a)
Oridonin 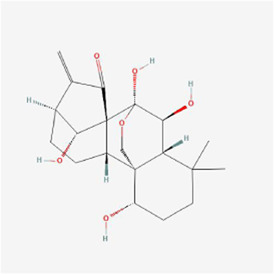	Rabdosia rubescens	Terpenes	Throat cancer	LSCC cells	ROS, CHOP and GRP78↑	Kang et al. (2020)
Guangsangon E	Leaves of Morus alba L	Diels-Alder adduct	Lung Cancer	A549 cells /female Balb/c nude mice	GRP78, IRE1α, ATF4 and ROS↑	Shu et al. (2021)
Fucoidan 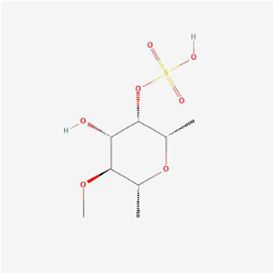	Brown seaweed	Polysaccharide	Lung Cancer	A549 and H1975 cells	GRP78 and its mediated caspase-3/PARP apoptosis pathway↑	Hsu et al. (2018)
Lathyrol 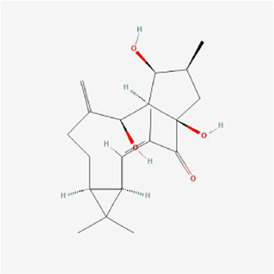	Semen Euphorbiae	Terpenes	Lung Cancer	A549, H460 cells and H460 cells were established as a subcutaneous lung cancer model in BALB/c nude mice	SERCA2↓,Ca2+ and GRP78↑, PERK-elF2α-ATF4-CHOP signal pathway↑	Chen et al. (2023)
*Dendrobium denneanum*	*Dendrobium denneanum Kerr*	Ether	Lung Cancer	A549 cells	disturbed the metabolic process of proteins and other substances, ERS↓	Zhang et al. (2019)
Kushenol Z	Sophora flavescens	Alcohols	Lung Cancer	A549 and NCI-H226 cells	CHOP, caspase-7/12↑	Chen et al. (2019)
Glycyrrhetinic acid 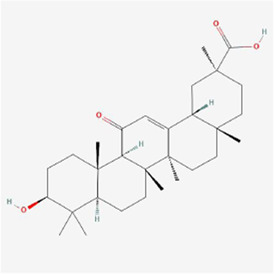	Liquorice	Terpenes	Lung Cancer	A549 and NCI-H460 cells	Bip, PERK and ERP72↑	Zhu et al. (2015)
*Arisema heterophyllum Blume*	Rhizoma Arisaematis	Agglutinin	Lung Cancer	A549 cells	p-eIF2α, CHOP, IRE1α and p-JNK↑	Feng et al. (2016)
Ginsenosides 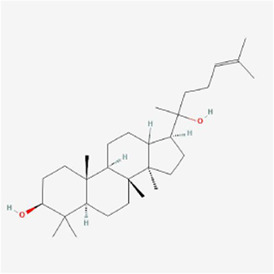	Ginseng (Panax ginseng C. A. Mey)	Glycosides	Lung Cancer	A549 cells	ATF4, CHOP, BIP↑, AKT-1 and p70 S6 kinase↓, ATG7↑	Zhao et al. (2019)
Evodiamine 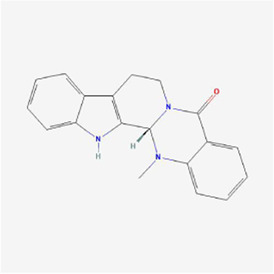	Evodia rutaecarpa (Juss.)	Quinazolines	Lung Cancer	Lewis lung cancer tumor-bearing mouse model/A549 and LLC cells	TRAF2, ASK1, p-JNK, caspase-12/9/3 and Bax↑,Bcl-2 ↓, mRNA expression of Grp78 and Ire1↑	Li et al. (2022b)
Flavonoid components in Astragali Radix	Astragali Radix	Flavonoid	Lung Cancer	Lewis lung cancer model in C57BL/6 mice	XBP1, IRE1 and GRP78↓, CHOP↑	Yang et al. (2019)
*Marsdenia tenacissima extract*	*Marsdenia tenacissima*	-	Lung Cancer	PC-9 and H1975 cells	ATF6, GRP-78, ATF4, xbp1, CHOP and ROS↑	Yuan et al. (2023a)
Corilagin 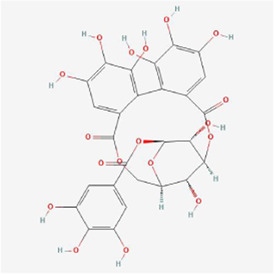	Phmllanthi Fructus	Phenol	Oesophagal cancer	ECA109 cells and KYSE150 cells	GRP78, ERS-induced cellular homeostasis↓, caspase-12/7 apoptotic signal pathways↑	Wu et al. (2021)
Daurisoline 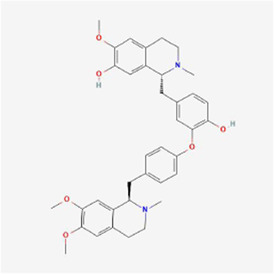	Menispermi	Alkaloids	oesophagal squamous cell carcinoma	EC1 and ECA109 cells	ROS and PERK-peIF2α-ATF4 signal pathway↑	Yuan et al. (2022b)
Tanshinone IIA 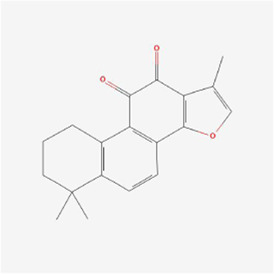	Salvia miltiorrhiza Bunge	Terpenes	Oesophagal cancer	Eca-109 cells	CHOP pathway↑	Zhang et al. (2017)
Betulinic Acid 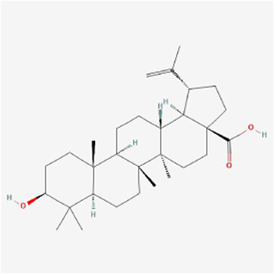	Birch bark	Terpenes	Breast cancer	MDA-MB-231 and BT-549 cells	GRP78↑, block GRP78 binding to PERK pathway, β-catenin/c-Myc pathway and glycolysis ↓	Zheng et al. (2019)
Dihydrotanshinone I 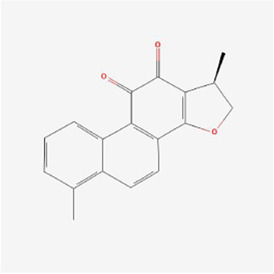	Salvia miltiorrhiza Bunge	Quinones	Breast cancer	MDA-MB-231 cells	ERp57↑, PERK-eIF2α-ATF4 apoptotic pathway↑	Shi et al. (2021)
Matrine 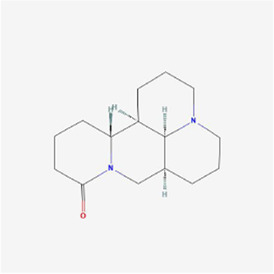	Sophora f lavescens	Alkaloids	Breast cancer	ER-7-positive MCF-7 cells	GRP78, eIF2α and CHOP↑	Xiao et al. (2017)
Peimine 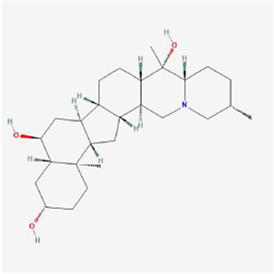	Fritillaria	Alkaloids	Breast cancer	MCF-7 cells	PERK, eIF2α and CHOP↓, ERS and NLRP3 inflammatory vesicles↓	Sun et al. (2023)
Schizandrin A 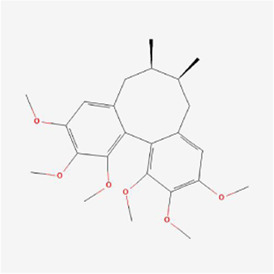	Fructus Schisandra	Benzene Derivatives	Stomach Cancer	AGS cells	GRP78, eIF2α, PERK and CHOP↑	Pu et al. (2021)
Curcumin 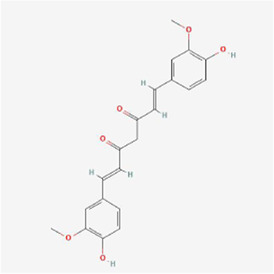	Rhizomes of Curcuma longa	Curdione	Stomach Cancer	AGS cells	CHOP and JNK pathways↑	Cao et al. (2013)
Wogonoside 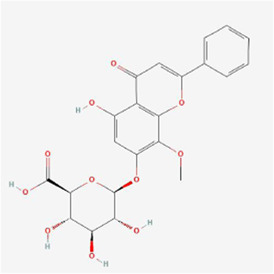	Scutellaria baicalensis	Sugar Acids	Stomach Cancer	AGS and MKN - 45 cells	GRP78, GRP94, IRE1α pathway, TRAF2, ASK1, JNK signaling pathway↑, Bax↑, Bcl-2↓	Gu et al. (2021)
Myristicin 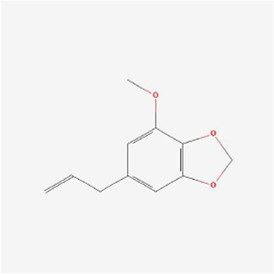	Myristica fragrans	Benzene Derivatives	Stomach Cancer	AGS and MKN - 45 cells	GRP78, phosphorylated IRE1α, PERK and ATF6↑, caspase-12↑	Song et al. (2023)
Isoquercitrin 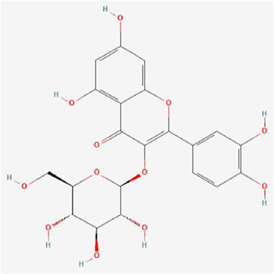	Hypericum perforatum L	Flavonols	Stomach Cancer	AGS and HGC-27 cells	p-PERK, p-eIF2α, GRP78 and CHOP↑, BCL-2↓, BAX↑, cleaved caspase-3/12↑	Liu et al. (2023)
KangFuXin	Periplaneta americana	Alcohols	Stomach Cancer	SGC-7901 cells	GRP78, CHOP and caspase-12↑	Chen et al. (2017)
The ethanolic extract of C. cicadae	Cordyceps cicadae	Ethanolic	Stomach Cancer	SGC-7901 cells	Ca2+, calpain-1, caspase-12/9↑	Xie et al. (2019)
Cytisine 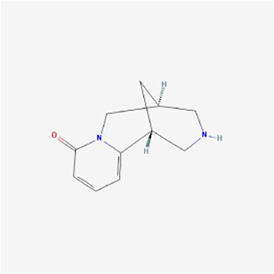	Sophora Alopecuraides L.	Alkaloids	Liver cancer	HepG2 cells	Ca2+, CHOP/GADD153, JNK and caspase-4↑	Yu et al. (2018)
Gecko polypeptide mixture	Gekko japonicus	polypeptide	Liver cancer	HepG2 cells	GRP78, ROS, PERK, ATF4, DDIT3, CHOP, PARP and caspase-3↑	Duan et al. (2018)
Muscone 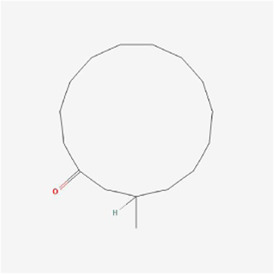	Musk	Cycloparaffins	Liver cancer	HepG2 cells	TRIB3, PERK, eIF2α, ATF4, DDIT3, caspase-3 and Bax ↑, Bcl-2↓	Qi et al. (2020)
Alisol B 23-acetate 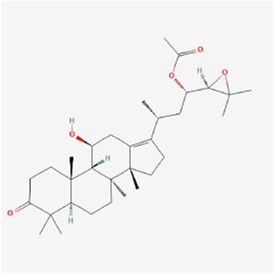	Alisma orientalis (Sam.) Juzep.	Steroids	Liver cancer	HepG2 cells	BIP, CHOP and intracellular Ca2+ ↑	Xu et al. (2015)
Realgar quantum dots	Realgar (As4S4)	-	Liver cancer	HepG2 cells	mRNA of Chop10 and GRP78↑	Qin et al. (2015)
Cryptomeridiol 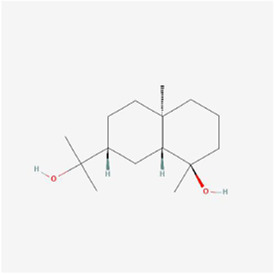	Magnolia Officinalis	Naphthalenes	Liver cancer	HepG2 cells /subcutaneous HepG2 xenografts in male BALB/c nude mice	IRE1α, ASK1, Bip, CHOP, Bkh126, JNK and p38↑	Li et al. (2022a)
Bufalin 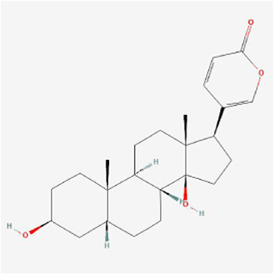	Bufonis Venenum	Glycosides	Liver cancer	HepG2 and Huh7 cells	IRE1-JNK pathway↑	Hu et al. (2014)
Bufalin 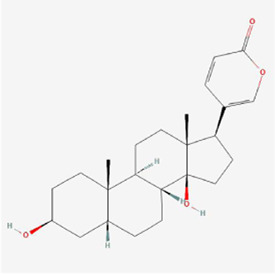	Bufonis Venenum	Glycosides	Liver cancer	HepG2 and Huh7 cells	eIF2α, CHOP and IRE1↑, p-Akt↓	Zhai et al. (2015)
Celastrol 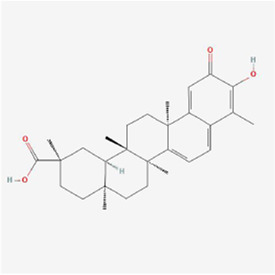	Tripterygium wilfordii and Celastrus regelii	Terpenes	Liver cancer	HepG2, Bel7402 cells /H22 tumor-bearing mice	GRP78/BiP, ATF4, CHOP, IRE1α and XBP1s↑	Ren et al. (2017)
Konjac glucomannan	Amorphophallus konjac K. Koch	Polysaccharide	Liver cancer	HepG2 and Bel-7402 cells	TLR4↓, PERK/ATF4/CHOP signaling pathway↑	Shi et al. (2022)
5,2’,4’-trihydroxy-6,7,5’-trimethoxyflavone-nps	Sorbaria sorbifolia	Flavonoid	Liver cancer	HepG2, Hep3B, and PLC/PRF/5 cells	GRP78, PERK, IRE1α, ATF6 pathways, CHOP and caspase4 ↑	Xiao et al. (2016)
Fisetin 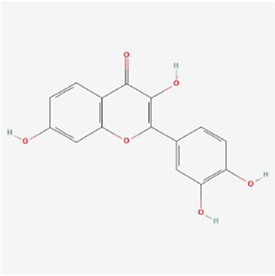	Traditional medicines, plants, vegetables, and fruits	Flavonols	Liver cancer	HepG2, Hep3B and Huh7 cells /HepG2 xenograft mouse models	Ca2+, CHOP, p-eIF2α, p-PERK, cleaved caspase-3, ATF4 and induction of GRP78 exosomes↑	Kim (2023)
Aqueous extract of *polygonum bistorta*	*Polygonum bistorta*	-	Liver cancer	Hep3B cells	ROS, DAPK3, caspase 8/9/3 and PARP 1 activity↑	Liu et al. (2017b)
Astragalus Polysaccharide 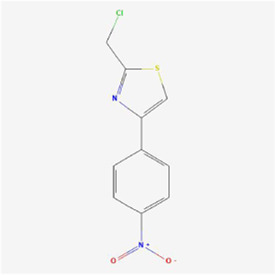	Astragalus membranaceus	Polysaccharide	Liver cancer	Hep3B cells	PERK/eIF2α/CHOP signaling pathway, caspase-3, Bax and Bim↑	Li et al. (2023)
Caudatin 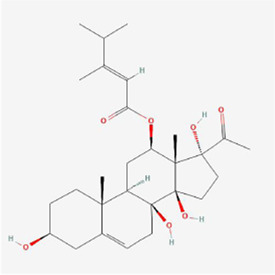	The roots of Cynanchum bungei Decne	Glycosides	Liver cancer	Diethylnitrosamine-induced hepatocellular carcinoma in a rat model	ATF6 and PERK-eIF2α-ATF4 pathways↓	Song et al. (2020)
Psoralen 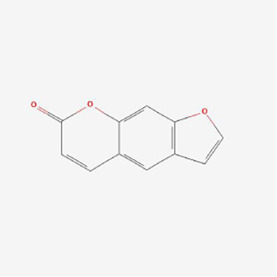	Psoralea corylifolia (L.)	Pyrans	Liver cancer	SMMC7721 cells	GRP78, GRP94, IRE1 and ATF6 pathways, DDIT3↑, caused endoplasmic reticulum Ca2+ disruption and Bcl-2↓	Wang et al. (2019)
Baicalein 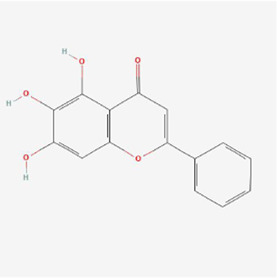	Scutellaria baicalensis Georgi	Flavonoid	Liver cancer	SMMC-7721 and Bel-7402 cells	PERK, IRE1 pathway, BiP, eIF2α and CHOP↑, Bcl-2, Bcl-xL and Mcl-1↓, caspase-9/3↑ and PARP↓	Wang et al. (2014)
Notopterol 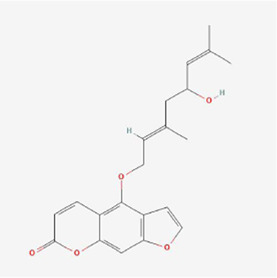	Notopterygium incisum	Pyrans	Liver cancer	HepJ5, Mahlavu cell line and female BALB/c nude mice	cancer stemness↓, PERK, CHOP and oxidative stress↑	Huang et al. (2023)
Lupeol 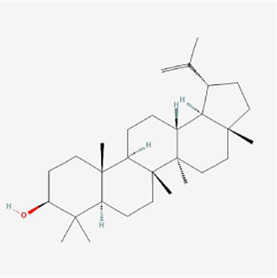	Edible fruits and vegetables	Terpenes	Colorectal cancer	LoVo Cells	eIF2α and caspase-3 phosphorylation↑	Chen et al. (2018)
Oridonin 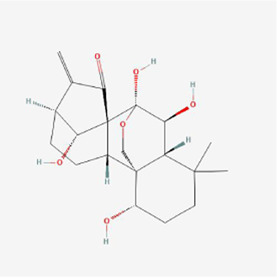	Rabdosia rubescens	Terpenes	Colorectal cancer	LoVo and RKO cells	TCF4↓, TP53, ATF4 and CHOP↑	Zhou et al. (2023)
Curcumin 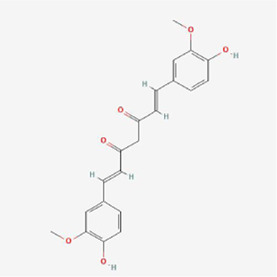	Rhizomes of Curcuma longa	Curdione	Colorectal cancer	LoVo and HT-29 cells	ROS, BIP, PDI and CHOP↑	Huang et al. (2017)
Curcumin 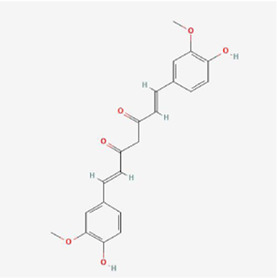	Rhizomes of Curcuma longa	Curdione	Colorectal cancer	HT-29 cells	CHOP and JNK pathways↑	Cao et al. (2013)
Osthole 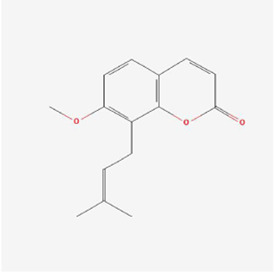	Cnidium monnieri	Pyrans	Colorectal cancer	HT-29 cells	GRP78, PERK, elF2α and CHOP↑	Zhou et al. (2021)
Artemisianins A	Artemisia argyi	Sesquiterpenoid dimers	Colorectal cancer	HT-29 cells	IRE1α, XBP1s, ATF6 ,CHOP, p-PERK and p-eIF2α↑, and disrupted intracellular Ca2+ homeostasis	Xue et al. (2019)
Palmitic acid 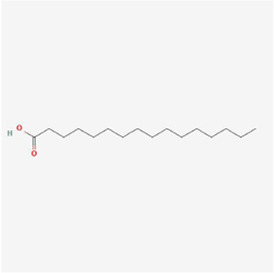	Palm oil and some animal products	Monounsaturated fatty acid	Colorectal cancer	HT-29 and HCT-116 cells	p-PERK, p-eIF2α, ATF4, Ca2+↑	Kuang et al. (2023)
A Purified Resin Glycoside Fraction	Pharbitidis Semen	Resin glycoside	Colorectal cancer	HT-29 and HCT-116 cells	BIP/GRP78, CHOP, IRE1α, XBP1s and ubiquitinated protein expression↑, proteasome-dependent degradation↓, MAPK signaling pathway↑, JNKs and ERKs↑	Zhu et al. (2019)
Periplogenin 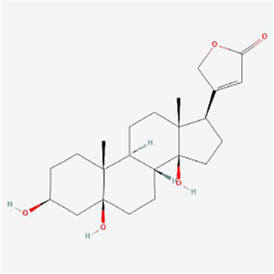	Periplocae	Glycosides	Colorectal cancer	HCT-116 cells	ROS, BIP, p-eIF2α, CHOP, IRE1α, p-JNK↑, p-ASK1↓, Bax↑, cleavage of caspase-3 and PARP↑, Bcl-2↓	Yang et al. (2021)
Shikonin 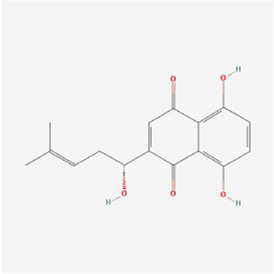	Lithospermum erythrorhizon	Quinones	Colorectal cancer	HCT-116 and HCT-15 cells /BALB/c nude mice	BiP, PERK/elF2α/ATF4/CHOP and IRE1α/JNK signaling pathways↑, Bcl-2 ↓, caspase-3/9↑	Qi et al. (2022)
Artesunate 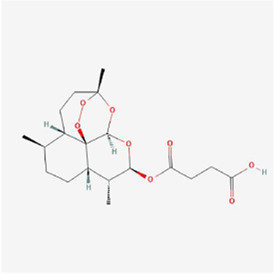	Artemisinin	Terpenes	Colorectal cancer	HCT-116, SW480 cells /BALB/c mice grown on CT-26 cells	BIP, IRE1 and p-IRE1α-CHOP-DR5 signaling pathway↑	Huang et al. (2022)
Ginsenoside Rg3 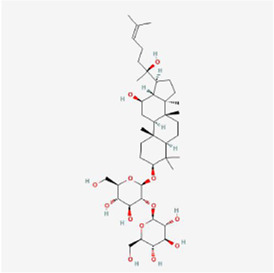	Ginseng	Glycosides	Gallbladder cancer	GBC-SD cells/ transplanted tumor-bearing mice	PERK, eIF2α, ATF4, CHOP, Lipocalin 2, ROS, lincRNA-p21↑	Wu et al. (2018)
Matrine 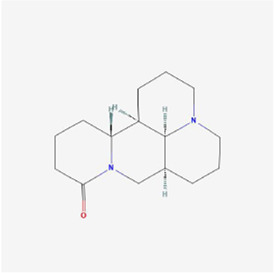	Sophora f lavescens	Alkaloids	Prostate cancer	DU145, PC-3 cells / DU145 cell xenografts in mice	Bip, PERK, eIF2α, ATF4, CHOP and Bax↑, Bcl-2↓	Chang et al. (2018)
Astragaloside 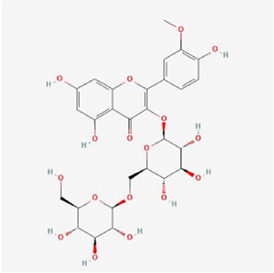	Astragalus membranaceus	Flavonoid	Prostate cancer	DU145 cells	BiP, IRE1, p-PERK, AFT6, AFT4, CHOP and caspase-12-related apoptotic pathways↑	Tan et al. (2018)
Isobavachalcone 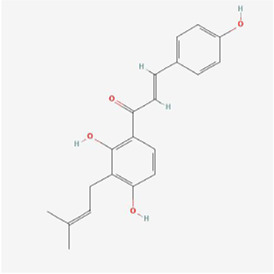	Psoralea corylifolia Linn.	Flavonoid	Prostate cancer	PC-3 cells	XBP-1, ATF4, GRP78, Chop and p-eIF2α proteins↑, TrxR1 protein↓, ROS ↑, and produced oxidative stress	Li et al. (2018)
Quercetin 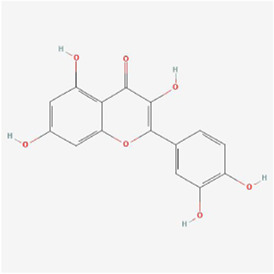	Many plants are found in stems, flowers, leaves, buds, seeds, fruits	Flavonols	Prostate cancer	PC-3 cells	Facilitate the transport of AIF protein released from mitochondria to the nucleus, ATF, GRP78 and GADD153↑	Liu et al. (2014)
Tanshinone IIA 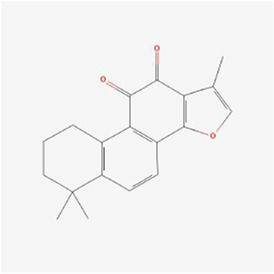	Salvia miltiorrhiza Bunge	Terpenes	Cervical cancer	CaSki cells	ROS, PERK, IRE1, eIF2α, ATF4 and CHOP levels ↑, led to PARP degradation, p38 and ASK signaling pathway↑	Pan et al. (2013)
Saikosaponin-A 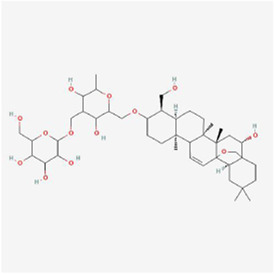	Bupleurum falcatum	Triterpenoids	Cervical cancer	HeLa cells /nude mice	GRP78, CHOP and caspase12↑	Du et al. (2021)
Celastrol 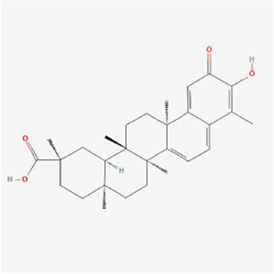	Tripterygium wilfordii and Celastrus regelii	Terpenes	Cervical cancer	HeLa cells	Bip, PERK and IRE1↑, and caused cytoplasmic vacuolization	Wang et al. (2012)
20(S)-Ginsenoside Rh2 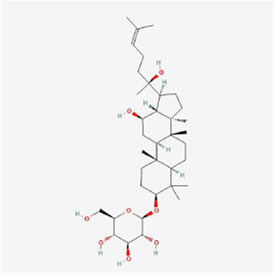	CelastrolGinseng	Glycosides	Cervical cancer	HeLa cells	ATF4, DDIT3, JUN, FOS, BBC3 and PMAIP1↑, DDIT3 heterodimer↓	Liu et al. (2022)
Myricetin 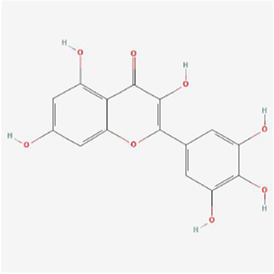	Walnuts, vegetables and fruits	Flavonoid	Ovarian cancer	SKOV3 cells	GRP78 and CHOP ↑	Xu et al. (2016)
Asperuloside 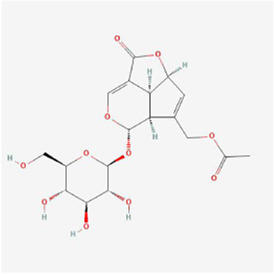	Herba Paederiae	Glycosides	Leukemia	U937 and HL-60 cells /primary leukemia cells (AML); tumor model for U937 cells xenograft in nude mice	GRP78, PERK, eIF2α, CHOP, p-IRE1, XBP1, ATF6 and cleaved caspase-12↑	Rong et al. (2020)
Shikonin 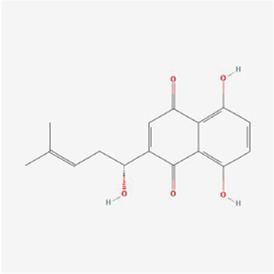	Lithospermum erythrorhizo	Quinones	Leukemia	ATLL cells	ROS, p-eIF2α, ATF4, XBP-1, p-JNK, and CHOP↑	Boonnate et al. (2023)
Polyphyllin I	Paris polyphylla Smith var.yunnanensis (Franch.) Hand.-Mazz	Glycosides	Osteosarcoma	MG-63, Saos-2 and U2OS cells	BiP, PERK-eIF2α-ATF4-GADD153 (CHOP) pathway↑, Bcl-2 and Bcl-xL↓, Bax and Bak↑, caspase-3 and PARP ↑	Chang et al. (2015)
(3R)-5,6,7-trihydroxy-3-isopropyl-3-methylisochroman-1-one 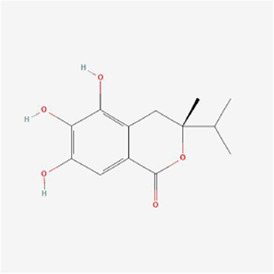	Selaginella moellendorffii Hieron.	Antioxidants	Osteosarcoma	U2OS cells and mice	IRE1, ATF6 and GRP78↑	Song et al. (2019)
Psoralen 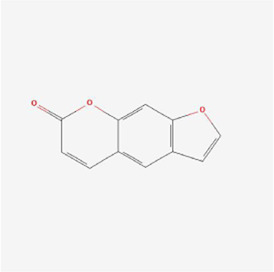	Psoralea corylifolia Linn. (Leguminosae)	Pyrans	Osteosarcoma	MG-63 and U2OS cells	GRP78, DDIT3, GADD34, EDEM1, GDF15 and S1P mRNA, CHOP, IRE1, XBP-1s, GRP78, PERK and ATF-6 ↑	Li and Tu (2022)
Shikonin 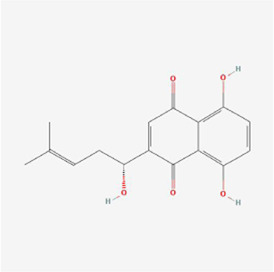	Lithospermum erythrorhizon	Quinones	Melanoma	A375 cells	ROS, p-eIF2α, CHOP and caspase-3↑	Liu et al. (2019)

## 4 Natural products target ERS-mediated apoptosis in Neuroglioma

Fatsioside A at 80 nM induced ERS in malignant glioma U87MG cells, causing apoptosis by enhancing the phosphorylation of PERK and eIF2α, upregulating CHOP protein level and promoting caspase-4 cleavage (Pan et al., 2015). The application of sinomenine hydrochloride at concentrations of 0.125 and 0.25 mM to human glioblastoma U87 and SF767 cells, respectively, led to a rise in the cytoplasmic levels of free Ca^2+^ and upregulated the expression of GRP78, PERK, IRE1, and CHOP; additionally, it suppressed the mesenchymal markers vimentin, Snail, and Slug, indicating that sinomenine hydrochloride reduces the invasive capacity of tumor cells by activating ERS and reversing epithelial-mesenchymal transition (EMT) (Jiang et al., 2018). In glioma U87MG cells and LN229 cells, bufalin triggered apoptosis via the mitochondrial and ERS pathways, and the mechanisms of ERS-mediated apoptosis included the upregulation of CHOP, ATF6 and GRP78, enhanced phosphorylation of PERK and eIF2α, and cleavage of caspase-4; bufalin also played a cytoprotective role through initiating the AMP-activated protein kinase (AMPK)/mammalian target of rapamycin (mTOR) pathway and PERK/eIF2α/CHOP pathway, forming acidic vesicular organelles, increasing autophagic lysosomes and accumulating LC3-II to induce cellular autophagy and thereby alleviating ERS, which suggested that ERS preceded bufalin-induced autophagy, that inhibition of autophagy strengthened its proapoptotic effect and that ERS could be engaged in apoptosis and autophagy simultaneously (Shen et al., 2014). In glioblastoma multiforme (GBM) cell lines and xenograft mouse models, isochaihulactone (K8) directly induced DNA damage inducible transcript 3 (DDIT3) activation and increased nonsteroidal anti-inflammatory drug-activated gene 1 expression, which was not dependent on GRP78 and PERK expression, and activated the caspase signaling pathway to upregulate caspase 3 expression, thereby disrupting endoplasmic reticulum homeostasis in GBM cell lines and inducing G2/M period cell cycle arrest and apoptosis (Tsai S. F. et al., 2017). In summary, the mechanism by which fatsioside A, sinomenine hydrochloride, and bufalin induce apoptosis in glioma cells is predominantly the PERK pathway, with U87MG cells being the primary cell line studied.

## 5 Natural products target ERS-mediated apoptosis in nasopharyngeal cancer

Guangsangon E at a concentration of 20 μM enhanced the expression of GRP78, IRE1α and ATF4 and reactive oxygen species (ROS) in the CNE1 human nasopharyngeal carcinoma cell line, activated the ERS pathway, along with boosted the level of cleaved caspase-3 and autophagy-related protein 5 (Atg5), thereby inducing apoptosis, and similar results were obtained *in vivo* using female BALB/c nude mice as a model (Shu et al., 2021). Gambogenic acid opened chloride channels and activated VSOR Cl^−^ channels, leading to the efflux of VSOR Cl^−^ currents, a decrease in the levels of ERS-related protein GRP78 and an increase in CHOP and ATF4 expression levels; the primary mechanism behind these processes was the overexpression of CHOP, which repressed the anti-apoptotic impact of the UPR and triggered apoptosis in the CNE-2Z cell line of nasopharyngeal carcinoma (Su et al., 2019). PP-22 triggered apoptosis by activating the ERS sensor PERK, which upregulated CHOP protein expression and upregulated BIP, protein disulfide isomerase (PDI), EROL-LA, and IRE-LA levels; additionally, PP-22 promoted apoptosis through the mitochondrial signaling apoptosis pathway, inhibited the extracellular regulated protein kinases (ERK) pathway, activated the p38-mitogen-activated protein kinase (MAPK) signaling pathway and downregulated the signal transducer and activator of transcription 3 pathway to trigger apoptosis in nasopharyngeal cancer CNE-2 cells; interestingly, PP-22 hindered apoptosis by promoting autophagy through ERK pathway inhibition (Tan et al., 2019). In nasopharyngeal cancer NPC-TW 039 cells, a concentration of 8 μM tetrandrine hindered cell viability and rendered G0/G1 period cell cycle arrest; it also induced apoptosis through a pathway involving calcium-mediated ERS and caspase, the mechanism of which was mainly due to the production of ROS and Ca^2+^ caused by powdered antibiotics, leading to intracytoplasmic Ca^2+^ release, ERS promotion, and enhanced expression levels of relevant proteins such as GADD153 and GRP78 (Liu K. C. et al., 2017). In summary, the mechanisms by which guangsangon E, gambogenic acid, PP-22, and tetrandrine induce apoptosis in nasopharyngeal cancer cells are predominantly the PERK and IRE1 pathways, with CNE cells being the primary cell line studied.

## 6 Natural products target ERS-mediated apoptosis in oral cancer

Glaucocalyxin A, at concentrations of 4 and 8 μM, activated the ERS-mediated PERK pathway in human oral squamous cell carcinoma SCC25 and CAL27 cells, promoted the ATF4-CHOP pathway and its downstream signal cation transport regulator-like protein 1 (CHAC1) expression, and increased ROS accumulation through degradation of glutathione, thereby promoting oxidative stress and inducing apoptosis (Wang et al., 2022). Arsenic-Trioxide at 0.5 µM in combination with 20 µM dithiothreitol caused apoptosis in oral cancer cells by the mechanism that arsenic-Trioxide in combination with dithiothreitol upregulated cytochrome c (Cyt C), BCL2-associated X (Bax) and Bak and decreased Bcl-2 and p53 levels, resulting in the loss of mitochondrial membrane potential (MMP), generated ROS-induced DNA strand breaks, and triggered apoptosis through the mitochondrial apoptotic pathway; additionally, it also caused ERS, increased GRP78, calpain 1 and calpain 2 levels and increased expression levels of the cysteine proteases caspase-3, -9 and -12 (Tsai C. W. et al., 2017). In summary, glaucocalyxin A and arsenic trioxide promoted apoptosis in oral cancer cells by modulating ERS.

## 7 Natural products target ERS-mediated apoptosis in throat cancer

Oridonin combined with cetuximab suppressed the proliferation of laryngeal squamous cell carcinoma (LSCC) cells with high expression of epidermal growth factor receptor (EGFR) through the phosphatidylinositol 3-kinase (PI3K)/protein kinase B (Akt) and Janus kinase/signal transducer and activator of transcription (STAT) pathway-regulated nuclear factor kappa-B (NF-κB) pathways while downregulating MMP and Bcl-2 expression, increasing Bax, caspase 9 cleavage and Cyt C levels, and triggering mitochondria-dependent apoptosis, while oridonin induced ROS production and upregulation of CHOP and GRP78 to trigger the ERS apoptotic pathway (Kang et al., 2020). It was evident that oridonin caused the death of laryngeal cancer cells by stimulating ERS.

## 8 Natural products target ERS-mediated apoptosis in lung cancer

Guangsangon E triggered apoptosis in non-small cell lung cancer (NSCLC) A549 cells through the ERS pathway, a finding also confirmed by *in vivo* experiments, and the mechanism of action was consistent with the anti-nasopharyngeal carcinoma pathway described above (Shu et al., 2021). Fucoidan promoted tunicamycin expression and activated the ERS pathway in lung cancer A549 and H1975 cells, which promoted apoptosis by upregulating the GRP78 protein and its mediated caspase-3/poly ADP-ribose polymerase (PARP) apoptotic pathway; additionally, fucoidan has been synergized with cisplatin to induce apoptosis and decrease the toxic effects of cisplatin by enhancing the viability of cisplatin-treated cells (Hsu et al., 2018). Lathyrol (30, 60 and 120 μg/mL) induced ERS and activated its mediated PERK-elF2α-ATF4-CHOP signaling pathway by downregulating Ca^2+^-ATPase 2 (SERCA2) activity in lung cancer A549 and H460 cells, increasing Ca^2+^ levels and GRP78 protein expression levels in the endoplasmic reticulum lumen, and inducing apoptosis by upregulating Bax, caspase-3 and Cyt C expression and downregulating anti-apoptotic Bcl-2 protein level; tumors were markedly inhibited by treatment with 10, 20 or 40 mg/kg lathyrol in a subcutaneous tumor model established in BALB/c nude mice with H460 cells (Chen et al., 2023). In NSCLC A549 cells, the ether extract of *Dendrobium denneanum* disrupted the metabolic processes of proteins and other substances, induced ERS reactions, disrupted cell self-repair mechanisms, and impaired cell adhesion and proliferation; moreover, *D. denneanum* notably elevated ROS levels in lung cancer cells and disturbed the intracellular redox system, causing lung cancer cells to initiate various processes, such as apoptosis and autophagy, to facilitate cell death (Zhang et al., 2019). In NSCLC A549 and NCI-H226 cells, kushenol Z exerted tumor suppressive effects by enhancing the Bax/Bcl-2 ratio and activating caspase-3/9, resulting in mitochondrial apoptosis while upregulating CHOP and activating caspase-7/12 expression to trigger the ERS apoptotic pathway; additionally, kushenol Z exerted anti-proliferative activity by inhibiting cAMP-phosphodiesterases and Akt expression from suppressing the mTOR pathway (Chen et al., 2019). Glycyrrhetinic acid did not trigger apoptosis in NSCLC A549 and NCI-H460 cells but stimulated cell cycle arrest in the G0/G1 period by enhancing cyclin-dependent kinase inhibitors (p18, p16, p27 and p21), suppressing cyclin (cyclin-D1, -D3 and -E) and cyclin-dependent kinases (CDK4, 6 and 2), maintaining retinoblastoma protein phosphorylation and suppressing E2F Transcription Factor 1 (E2F1) in both cell lines, and significantly increasing the expression levels of Bip, PERK, and endoplasmic reticulum resident protein 72 (ERP72), regulatory proteins associated with ERS, which might be the mechanism by which glycyrrhetinic acid inhibits NSCLC cell proliferation (Zhu et al., 2015). *Arisema heterophyllum* Blume (AHA) curbed the propagation of lung cancer A549 cells, caused cell cycle arrest in the G1 period, and affected apoptosis by restraining the PI3K/Akt pathway and inducing ERS through the mechanism of enhancement of Bax, reduction of Bcl-2, and initiation of caspase-9/3; additionally, AHA elevated the levels of p-eIF2α, CHOP, IRE1α and p-JNK to promote ERS, and increased LC3-II and Autophagy Related Protein 7 (ATG7) expression to induce autophagy (Feng et al., 2016). In NSCLC A549 cells, extracts of total ginsenosides upregulated the protein expression of ATF4, CHOP and BIP, inhibited AKT-1 and p70 S6 kinase phosphorylation, increased ATG7 expression levels, activated ERS, promoted LC3-II levels and autophagosome formation, and increased autophagic flux, thereby inducing cell autophagic death, a process that is mediated via the ATF4-CHOP-AKT1-mTOR axis (Zhao et al., 2019). Evodiamine upregulated GRP78 protein expression in NSCLC LLC and A549 cell lines and LLC tumor-bearing mouse models, activated ERS pathway-mediated autophosphorylation of IRE1, which then bound to TRAF2 and attracted ASK1 to constitute the IRE1-TRAF2-ASK1 compound, activated the JNK pathway, upregulated caspase-12/9/3 and Bax expression and downregulated Bcl-2 levels to induce associated apoptosis (Li Y. et al., 2022). In the Lewis lung cancer model in C57BL/6 mice, flavonoid components in Astragali Radix showed significant inhibition of tumor growth in both the low (5 g/kg/d) and high (10 g/kg/d) dose groups, while the high dose group lowered the expression of XBP1, IRE1 and GRP78 and enhanced the expression of CHOP, which subsequently promoted the ERS apoptotic pathway and mediated the apoptosis of tumor cells (Yang et al., 2019). *Marsdenia tenacissima* extract (MTE) at a concentration of 40 mg crude/mL notably upregulated the expression of ATF6, GRP-78, ATF4, xbp1, and CHOP in NSCLC PC-9 and H1975 cells, activated ERS while increasing ROS production, and upregulated immunogenic cell expression of death-related markers (ATP, HMGB1) and reduced MMP, thereby inducing apoptosis; in addition, MTE was shown to trigger ERS and immunogenic cell death by inhibiting tyrosine protein kinase receptor AXL activity (Yuan et al., 2023a). In summary, the mechanisms by which guangsangon E, fucoidan, lathyrol, and others induce apoptosis in lung cancer cells are predominantly the PERK and IRE1 pathways, with A549 cells being the primary cell line studied.

## 9 Natural products target ERS-mediated apoptosis in oesophageal cancer

Corilagin caused programmed cell death in ECA109 and KYSE150 oesophageal cancer cells, which occurred by reducing the levels of GRP78 and disrupting the cellular balance induced by ERS; simultaneously, corilagin enhanced the initiation of the cleaved caspase-12/7 pathways to evoke apoptosis; in addition, corilagin also promoted apoptosis and cell migration by activating the mitochondria-associated apoptotic signaling pathway (Wu et al., 2021). Daurisoline induced cell death in EC1 and ECA109 cells of oesophageal squamous cell carcinoma, mainly because daurisoline resulted in the aggregation of ROS in the cells and induced the initiation of the ERS-mediated PERK-peIF2α-ATF4 signaling pathway, and when Noxa was activated as one of the downstream targets of ATF4, it bound to myeloid cell leukemia-1 (Mcl-1), an anti-apoptotic protein in the Bcl-2 family, triggered the expression of cleaved caspases 9, 3 and 7 and activated the endogenous apoptotic pathway to promote apoptosis; in contrast, ATF4 upregulated CHOP expression and activated the death receptor 5 (DR5) recombinant protein, followed by the upregulation of the exogenous apoptosis-related marker cleaved caspase 8 and its downstream-mediated truncated bid gene to activate the exogenous apoptotic pathway to promote apoptosis (Yuan S. et al., 2022). Apoptosis was observed in Eca-109 cells of human oesophageal cancer when exposed to tanshinone IIA at a concentration of 60 μg/mL, which was achieved in two primary ways: first, by promoting the level of Cyt C and initiating caspase-9/3, thereby cleaving PARP and leading to apoptosis; second, by activating the ERS-mediated CHOP pathway after downregulating the expression of binding immunoglobulin protein, thereby inducing apoptosis in Eca-109 cells (Zhang et al., 2017). In summary, corilagin, daurisoline, and tanshinone IIA could regulate ERS to promote apoptosis in oesophageal cancer cells, and they worked primarily through the ECA109 cell line.

## 10 Natural products target ERS-mediated apoptosis in breast cancer

Betulinic acid at 20 and 40 μM upregulated the expression of GRP78 in breast cancer MDA-MB-231 and BT-549 cells, triggered the ERS apoptotic pathway, induced cell mortality, blocked the binding of GRP78 to the PERK pathway and suppressed the β-catenin/c-Myc pathway to suppress cell metastasis and aggression; moreover, betulinic acid decreased the levels of N-cadherin and vimentin, which are mesenchymal markers, and upregulated the epithelial marker E-cadherin, which demonstrated that betulinic acid inhibited EMT and significantly reduced the release of matrix metalloproteinase-2 and matrix metalloproteinase-9, thereby attenuating the metastasis and aggression of breast cancer cells (Zheng et al., 2019). Dihydrotanshinone I significantly inhibited endoplasmic reticulum resident protein 57 (ERP57), a chaperone protein that mediates the correct folding of endoplasmic reticulum glycoproteins in MDA-MB-231 cells, which activated ERS; the unfolded protein bound to BiP and separated it from PERK, which initiated PERK and drove eIF2α phosphorylation, leading to the upregulated expression of ATF4 and ultimately to apoptosis (Shi et al., 2021). Matrine induced apoptosis in breast cancer ER-7-positive MCF-7 cells via two main pathways: firstly, matrine enhanced the levels of GRP78, eIF2α, and CHOP, activating ERS and facilitating apoptosis; secondly, matrine downregulated the expression of intracellular hexokinase-II, reduced mitochondrial ATP production and inhibited energy metabolism, thus promoting apoptosis (Xiao et al., 2017). ERS is actively expressed in breast cancer cells and is involved in the initiation of NOD-like receptor pyrin domain-containing protein 3 (NLRP3), and 10 μM peiminine markedly reduces the expression levels of PERK, eIF2α and CHOP in breast cancer MCF-7 cells, suggesting that ERS is inhibited while blocking the activation of NOD-like receptor pyrindomain-containing protein3 (NLRP3) inflammatory vesicles, thereby inhibiting cell growth and inducing apoptosis (Sun et al., 2023). In summary, betulinic acid and dihydrotanshinone I mainly affected the MDA-MB-231 cells, and matrine and peiminine could affect the MCF-7 cells; they primarily promoted the apoptosis of breast cancer cells through the PERK pathway.

## 11 Natural products target ERS-mediated apoptosis in stomach cancer

Schizandrin A at 30 μM notably enhanced the level of the ERS receptor GRP78 protein in AGS gastric cancer cells and caused apoptosis by inducing phosphorylation of the eIF2α and PERK signaling pathways, along with upregulating the level of CHOP and promoting the ERS-mediated apoptosis pathway (Pu et al., 2021). In AGS gastric cancer cells, curcumin initiated ERS and enhanced the activation of the CHOP and Jnk pathways; moreover, curcumin increased the Ca^2+^ concentration in cells, stimulated the release of mitochondrial Cyt C, and caused apoptosis by downregulating MMP and Bcl-2 expression in cells; the half maximal inhibitory concentration (IC_50_) was 21.9 ± 0.1 μM in AGS cell lines (Cao et al., 2013). Wogonoside at 50 μM resulted in the activation of ERS in gastric cancer AGS and MKN-45 cells, increased levels of GRP78 and glucose regulated protein 94 (GRP94) proteins and activated the IRE1α pathway and its mediated TRAF2 and ASK1-related complexes, thereby activating the JNK signaling pathway and inducing apoptosis by enhancing Bax expression and reducing Bcl-2 expression (Gu et al., 2021). MKN-45 and AGS gastric cancer cell lines were treated with 7.8125, 15.625 and 31.25 μM myristicin, and the findings indicated that the expression of GRP78, the connector protein of ERS, phosphorylated IRE1α and PERK, and ATF6 were increased, while Myristicin upregulated caspase-12, the executor protein of ERS-triggered cell death, and triggered apoptosis (Song et al., 2023). Isoquercitrin at concentrations of 20–80 μM significantly elevated the levels of p-PERK, p-eIF2α, GRP78 and CHOP in gastric cancer HGC-27 and AGS cells while downregulating BCL-2 expression, upregulating BAX expression and cleaved caspase-3/12, and activating the ERS pathway; moreover, isoquercitrin enhanced the level of the immunogenic cell death (ICD) markers ATP, high-mobility group Box 1 (HMGB1), HSP70 and HSP90, promoting ICD and disrupting the immunosuppression of gastric cancer cells, thereby inducing cell death (Liu et al., 2023). Kangfuxin at 1 μg/mL was able to promote GRP78 protein expression, accelerate ERS, upregulate CHOP and caspase-12 protein concentration, and increase the release of autophagy-related proteins Beclin-1 and LC3-II/LC3-I in gastric cancer SGC-7901 cells; the trend of ERS-induced apoptosis and cellular autophagy was the same, and cellular autophagy could not be activated even after ERS was inhibited, so it can be concluded that Kangfuxin promotes apoptosis by activating autophagy, which was carried out after promoting ERS (Chen et al., 2017). The ethanolic extract of *C. cicadae* (EEC), on the one hand, upregulated the expression of Bax, AIF, caspase-8/6/3, decreased Bcl-2 and MMP levels in gastric cancer SGC-7901 cells, promoted the release of Cyt C from mitochondria, activated the cell surface receptors Fas and cleaved PARP, causing mitochondrial dysfunction and inducing the mitochondrial apoptosis pathway; otherwise, EEC upregulated the expression of the transcription factors E2F1, cyclin A2, cyclin E and p53 and downregulated CDK2 expression to block the cell cycle in S phase; additionally, EEC enhanced the intracellular Ca^2+^ concentration, promoted Ca^2+^ release from the endoplasmic reticulum and upregulated the expression of the ERS apoptosis pathway-related proteins calpain-1 and caspase-12/9 (Xie et al., 2019). In summary, the mechanisms by which schizandrin A, curcumin, wogonoside, and others induce apoptosis in stomach cancer cells are mainly the PERK and IRE1 pathways, and the AGS cells are the primary cell line that has been studied.

## 12 Natural products target ERS-mediated apoptosis in liver cancer

Cytisine at a concentration of 10 mmol/L significantly increased the Ca^2+^ concentration in liver cancer HepG2 cells, disrupted endoplasmic reticulum Ca^2+^ homeostasis and caused ERS, upregulated CHOP/GADD153, JNK and caspase-4, and finally activated the corresponding pathway to trigger apoptosis (Yu et al., 2018). Gecko polypeptide mixture at 0.3 mg/mL upregulated GRP78 protein expression and induced ROS production in hepatocellular carcinoma HepG2 cells, activated the ERS-mediated PERK signaling pathway and upregulated ATF4 expression to promote DDIT3 and CHOP expression in the cells, ultimately inducing apoptosis through starting of the apoptosis-associated proteins PARP and caspase-3 (Duan et al., 2018). Muscone notably elevated the expression of Tribbles pseudokinase 3 (TRIB3) in hepatocellular carcinoma HepG2 cells with an IC_50_ of 0.663 μM; Muscone activated the ERS-mediated PERK pathway and subsequently induced eIF2α phosphorylation and complex formation with ATF4, induced DDIT3 expression, upregulated caspase-3 and Bax expression and lowered Bcl-2 expression to induce apoptosis; additionally, muscone activated Sestrin 2, an autophagy-related gene, and AMPK to inhibit intracellular mTOR expression, thereby inducing cellular autophagy (Qi et al., 2020). The IC_50_ of alisol B 23-acetate isolated from *Alisma orientalis* (Sam.) Juzep. was 18.01 µM, and alisol B 23-acetate upregulated the expression of ERS markers BIP and CHOP in HepG2 cells in a concentration-dependent manner while increasing the intracellular calcium concentration and activating ERS to suppress cell growth and promote apoptosis; additionally, alisol B 23-acetate also upregulated the expression of LC3II to promote autophagy (Xu et al., 2015). Realgar quantum dots (RQDs) at 30 μg/mL declined Bcl-2 expression and elevated Bax expression in hepatocellular carcinoma HepG2 cells, promoted Cyt C release with complete loss of MMP and triggered an apoptotic enzyme cascade effect, together with the upregulated mRNA expression of Chop10 and GRP78, which suggested that RQDs induced apoptosis and necrosis probably via ERS, loss of MMP, and an elevated Bax/Bcl-2 ratio (Qin et al., 2015). In liver cancer cells and male BALB/c nude mice xenografted with subcutaneous HepG2 cells, cryptomeridiol stimulated the IRE1α-ASK1-JNK signaling pathway and induced MMP loss by translocating the orphan nuclear receptor Nur77 to mitochondria, resulting in mitochondrial dysfunction, while cryptomeridiol activated IRE1α and ASK1, stimulated IRE1α and PERK phosphorylation, and significantly upregulated Bip and CHOP expression; since ASK1 mediates IRE1α signaling, Bkh126 could promote ASK1 activity and subsequently increase the phosphorylation of JNK and p38 (both substrates and effectors of ASK1) to stimulate the ERS signaling pathway; ERS exacerbation and mitochondrial dysfunction led to increased ROS cytotoxic products and induced apoptosis, suggesting that Bkh126 induced Nur77 to link ERS to mitochondria-mediated cell killing (Li X. et al., 2022). Bufalin at 80 nmol/L activated the ERS-mediated IRE1-JNK pathway in HCC Huh-7 and HepG-2 cells and induced apoptosis, but subsequent studies showed that IRE1 upregulated Beclin-1 and autophagy-associated protein Atg5 levels, promoted the transformation of LC3-I to LC3-II, inhibited p62 expression, and triggered cytoprotective autophagy to counteract apoptosis (Hu et al., 2014). The administration of bufalin to HepG2 and Huh7 hepatocellular carcinoma cells exhibited a synergistic inhibition of tumor cell growth and triggered apoptosis when combined with sorafenib, which effectively reversed both intrinsic and acquired resistance to sorafenib through various mechanisms; specifically, the activation of Akt by sorafenib and subsequent resistance were counteracted by bufalin’s time-dependent upregulation of eIF2α, CHOP, and IRE1 expression; moreover, bufalin induced Akt inactivation by reducing phosphorylated Akt levels through the IRE1 pathway in an ERS-dependent manner (Zhai et al., 2015). Celastrol inhibited the reproduction of liver cancer HepG2 and Bel7402 cells, causing G2/M cell cycle arrest; following a 24-h treatment of HepG2 cells with 0.625–10 μM celastrol, GRP78/BiP, ATF4, CHOP, IRE1α and XBP1 splice forms (XBP1s) in the cells were significantly increased, and immunoblotting results showed that celastrol enhanced phosphorylation of IRE1α and xbp1, particularly at 2.5 μM, which suggested that celastrol caused ERS in hepatocellular carcinoma cells (HCC); in a homozygous model study of H22 tumor-bearing mice, GRP78/BiP expression was upregulated, and caspase-3 was activated in celastrol-treated mice, suggesting that celastrol enhanced ERS and caspase activity inhibition of tumor growth (Ren et al., 2017). Compared with Konjac glucomannan (KGM) or 5-Fluorouracil (5-FU) alone, KGM combined with 5-FU notably induced apoptosis and suppressed cell proliferation and migration with inhibition of Toll-like receptor 4 (TLR4) expression to activate ERS and its mediated PERK/ATF4/CHOP signaling pathway in HCC HepG2 and Bel-7402 cells; moreover, KGM reversed the resistance of HCC to 5-FU by decreasing TLR4 *in vivo* experiments (Shi et al., 2022). Nanoparticles (TTF1-nps) of TTF1 (5,2′,4′-trihydroxy-6,7,5′-trimethoxyflavone) upregulated the expression of GRP78 in liver cancer lines (HepG2, Hep3B, PLC/PRF/5), activated three main pathways involved in ERS, namely, PERK, IRE1α, and ATF6, and upregulated the expression of CHOP and caspase4, thereby inducing apoptosis, and TTF1-nps at 5, 10 and 20 μmol/kg doses treated with nude mouse HepG2 xenograft models for 16, 18 and 20 days, respectively, were also shown to suppress the proliferation of tumor cells (Xiao et al., 2016). Fisetin (3,7,3′,4′-tetrahydroxyflavone), in HCC HepG2, Hep3B and Huh7 cells and HepG2 xenograft mouse models, promoted apoptosis by releasing Ca^2+^, increasing CHOP, p-eIF2α, p-PERK, cleaved caspase-3, ATF4 and induction of GRP78 exosomes to activate the ERS-mediated PERK-ATF4-CHOP signaling pathway; moreover, ERS inhibited the EMT phenomenon under radiation conditions and reversed radiotherapy resistance (Kim, 2023). Aqueous extract of *Polygonum bistorta* (PB) at 240 μg/mL upregulated the expression of LC3B-II protein, GFP-LC3B puncta and p62/Sequestosome 1, a marker of autophagosome formation in hepatocellular carcinoma Hep3B cells, suggesting that PB enhanced the aggregation of cellular autophagosomes, and some studies claim that excessive accumulation of autophagy can induce apoptosis; moreover, PB accelerated the creation of ROS and upregulated the expression of death-associated protein kinase 3 (DAPK3), an upstream integrator of ERS-mediated apoptosis, and the expression of caspase 8/9/3 and PARP 1 activity was notably upregulated, confirming the pro-apoptotic effect of ERS in Hep3B cells; similar results were obtained in Hep3B and HepG2 xenograft mouse models after 276 mg/kg PB treatment (Liu Y. H. et al., 2017). Treatment with 50 mg/L Astragalus polysaccharide combined with 1 μM doxorubicin lowered the expression of O-GlcNAc transferase and enhanced the expression of O-GlcNAcase in hepatocellular carcinoma Hep3B cells, which ultimately inhibited O-GlcNAcylation in Hep3B cells, thereby promoting doxorubicin-induced ERS expression, activating the PERK/eIF2α/CHOP signaling pathway, and upregulating cleaved caspase-3, Bax and Bcl-2 interacting mediator of cell death (Bim) expression by inhibiting Bcl-2 expression, thus inducing Hep3B cell apoptosis; similar experimental results were obtained *in vivo* with 50 mg/kg Astragalus polysaccharide and/or 2 mg/kg doxorubicin treatment in Hep3B mouse xenograft tumor models (Li et al., 2023). In a rat model of diethylnitrosamine (DEN)-induced hepatocellular carcinoma, caudatin at 50 mg/kg modulated ERS in rat cells and triggered apoptosis in hepatocellular carcinoma cells by suppressing the ATF6 and PERK-eIF2α-ATF4 pathways, while caudatin reduced interleukin-6, macrophage chemoattractant protein-1 and interleukin-1β levels and biomarkers of liver inflammation, which were effective in the prevention of liver cancer (Song et al., 2020). Psoralen at 40 μM promoted apoptosis in liver cancer SMMC7721 cells by enhancing GRP78 and GRP94 protein expression, activating the IRE1 and ATF6 pathways, and improving the gene expression of DDIT3, resulting in disruption of endoplasmic reticulum Ca^2+^ homeostasis and downregulating the expression of Bcl-2 (Wang et al., 2019). Baicalein at a concentration of 200 μM upregulated BiP expression in hepatoma SMMC-7721 and Bel-7402 cells, activated PERK and IRE1 pathways and the phosphorylation of UPR downstream molecules CHOP and eIF2α, decreased the expression of Bcl-2, Bcl-xL and Mcl-1, activated caspase-9/3 and inhibited the DNA repair enzyme PARP to induce apoptosis; moreover, baicalein increased the conversion of LC-3I to LC-3II and promoted apoptosis by increasing cellular autophagy (Wang et al., 2014). Notopterol, which exerted antitumor effects through inhibition of cancer stemness, upregulation of PERK and CHOP, enhancement of ERS and increased oxidative stress in HCC HepJ5, Mahlavu cell line and female BALB/c nude mice (Huang et al., 2023). In summary, the mechanisms by which cytisine, gecko polypeptide mixture, muscone, and others induce apoptosis in liver cancer cells are mainly the PERK, IRE1, and ATF6 pathways, and HepG2 cells are the primary cell line that has been studied.

## 13 Natural products target ERS-mediated apoptosis in colorectal cancer

Overexpression of ATP-binding cassette, subfamily G (WHITE), member 2 (ABCG2/BCRP) led to resistance of tumor cells to multiple chemotherapeutic agents; after lupeol treatment, ABCG2 expression was downregulated in the LoVo colorectal cancer cell and the oxaliplatin-resistant OXA-R LoVo cell, but ERS marker levels as well as eIF2α (P-eIF2α) and caspase-3 phosphorylation levels were significantly increased, suggesting that lupeol might induce apoptosis by inhibiting ABCG2 and subsequently activating the ERS pathway, as was the case in the tumor-bearing mice; moreover, cell viability of the OXA-R LoVo cell line was significantly reduced after 50 and 100 μM lupeol treatment compared to that of the parental LoVo cell line, and apoptosis was increased after 24 h of 50 μM lupeol treatment in the OXA-R LoVo cell line, thus lupeol has the prospective to be a valid treatment for chemotherapy multidrug resistant cancers, particularly in oxaliplatin-resistant LoVo CRC cells (Chen et al., 2018). Transcription factor 4 (TCF4) was a key regulator of ERS, and tumor protein p53 (TP53) could inhibit TCF4 activity; in CRC LoVo and RKO cells, oridonin upregulated the expression of TP53 in a concentration-dependent manner, which inhibited the activity of TCF4 and caused the cellular ROS aggregation and disrupting Ca^2+^ homeostasis, and elevating the expression levels of ATF4 and CHOP, which ultimately led to apoptosis; this suggested that Oridonin exerts its antitumor effects by regulating ERS induced via the TP53/TCF4 axis (Zhou et al., 2023). Curcumin triggered apoptosis and G0/G1 cycle arrest by heightening intracellular calcium levels in CRC LoVo and HT-29 cells, while curcumin and/or irinotecan produced ROS and elevated the expression levels of ERS-related proteins BIP, PDI and CHOP to activate the apoptotic pathway of the endoplasmic reticulum, which large amounts of ROS can further activate; thus, curcumin improved the ability of irinotecan to facilitate apoptosis in CRC cells by producing ROS and activating the ERS pathway (Huang et al., 2017). Curcumin exhibited an IC_50_ value of 40.7 ± 0.5 μM, which led to the initiation of the ERS pathway and upregulated the expression of CHOP and jnk pathways in CRC HT-29 cells; moreover, curcumin could increase the concentration of Ca^2+^ in cells, facilitating the liberation of mitochondrial Cyt C and ultimately decreasing MMP and Bcl-2 expression levels and upregulating bax expression to induce apoptosis (Cao et al., 2013). Osthole effectively upregulated the expression of GRP78, PERK, elF2α and CHOP proteins in colorectal cancer HT-29 cells at both 25 and 50 µM concentrations, activating ERS and promoting apoptosis, inducing autophagy and thus promoting apoptosis by upregulating the LC3-II/LC3-I ratio and downregulating p62 protein expression (Zhou et al., 2021). Artemisianins A exhibited a strong repressive effect on CRC HT-29 cells with an IC_50_ value of 7.2 μM, and it increased the expression of IRE1α and XBP1s, upregulated autophosphorylated p-PERK and PERK-mediated p-eIF2α phosphorylation, and upregulated the expression of ATF6 and CHOP, suggesting that artemisinin A triggered apoptosis in HT-29 cells and activated ERS apoptotic signaling for tumor suppression by upregulating the UPR pathway of IRE1α, p-PERK, ATF6 and CHOP and by disrupting intracellular Ca^2+^ homeostasis (Xue et al., 2019). Palmitic acid at a dose of 120 μmol/L upregulated the expression of p-PERK, p-eIF2α and ATF4 in CRC HT29 and HCT116 cell lines, which promoted the translocation of transferrin by activating Ca^2+^ release from ERS, thereby inducing ferroptosis caused by iron accumulation and eventually cell death, while high expression of fatty acid translocase (FAT/CD36) facilitated the enhancement of palmitic acid-induced ferroptosis; BALB/c-nu mouse models of HT29 cells, SW620 cells and SW620 cells with high CD36 expression were established, and after treatment with 10 mg/kg palmitic acid in an *in vitro* experiment, the effects were in general agreement with the *in vivo* experiment (Kuang et al., 2023). A purified resin glycoside fraction from Pharbitidis Semen (RFP)-induced apoptosis was mediated by the coexistence of caspase independence and autophagy protection and characterised by cytoplasmic vacuole accumulation and mitochondrial swelling; this compound triggered paraptosis-like apoptosis by upregulating the ERS markers BIP/GRP78, CHOP, IRE1α, XBP1s and ubiquitinated proteins, activating the IRE1 branch of UPR signaling, suppressing proteasome-dependent degradation and activating the MAPK signaling pathway, and RFP-induced cytoplasmic vacuolisation and apoptosis were positively modulated by JNKs and ERKs, which were not associated with ROS generation or intracellular calcium stabilisation; furthermore, RFP was found to arouse intracellular Chloride Intracellular Channel Protein 1 (CLIC1) and increase intracellular CI^−^ concentrations, as blocking CLIC1 attenuated cell death, cytoplasmic vacuolation and ERS, and therefore the cytotoxic effect of RFP on CRC HT-29 and HCT-116 cells was caused by CLIC1-mediated apoptosis (Zhu et al., 2019). ROS are an upstream regulator of ERS, and ROS-ERS is an important pathway for apoptosis induction by natural products; the IC_50_ values of 10.5 and 2.8 µM after 24 and 48 h of treatment with periplogenin produced ROS, increased BIP, p-eIF2α, CHOP, IRE1α, and p-JNK protein levels and decreased p-ASK1 protein levels, suggesting that periplogenin simultaneously activated two signaling pathways in ROS-mediated ERS, BIP-eIF2α-CHOP and IRE1α-ASK1-JNK; moreover, this compound upregulated Bax expression and downregulated Bcl-2 expression, lowered the cleavage of caspase-3 and PARP, and promoted cell apoptosis, while periplogenin exhibited dose- and time-dependent inhibition of the viability of CRC HCT116 cells (Yang et al., 2021). Shikonin at 1.5 μM significantly upregulated BiP protein expression in CRC HCT-116 and HCT-15 cells, activated the ERS-mediated PERK/elF2α/ATF4/CHOP and IRE1α/JNK signaling pathways, lowered the expression of Bcl-2 and enhanced the expression of caspase-3/9 to trigger apoptosis in relevant cells; the same results were obtained after 3 mg/kg shikonin in HCT-116 and HCT-15 cell-grown rhabdoid mice (Qi et al., 2022). Artesunate at concentrations of 1–4 μM promoted the accumulation of ROS in CRC SW480 and HCT116 cells and enhanced the expression of the cellular senescence-associated proteins p16, p21, IL-6 and MMP3, leading to cellular senescence and cell cycle arrest; the upregulated expression of BIP and IRE1 showed that artesunate promoted the expression of the ERS mechanism, which mediated the activation of the p-IRE1α-CHOP-DR5 signaling pathway; similarly, *in vivo* experiments showed that the growth of tumors was inhibited after artesunate treatment in tumor-bearing mice grown with CRC CT26 cells (Huang et al., 2022). In summary, the mechanisms by which lupeol, oridonin, curcumin, and others induce apoptosis in colorectal cancer cells are predominantly the PERK and IRE1 pathways, with HT-29 and HCT-116 cells being the main cell lines studied.

## 14 Natural products target ERS-mediated apoptosis in gallbladder cancer

Ginsenoside Rg3 stimulated the ERS-mediated PERK pathway, leading to the increased phosphorylation of eIF2α, ATF4, CHOP, and Lipocalin 2 in GBC-SD gallbladder cancer cells and transplanted tumor-bearing mice; this activation also induced ROS production and significantly upregulated the expression of lincRNA-p21, which suggested that Rg3 suppressed propagation and enhanced apoptosis by promoting the apoptotic signaling pathway mediated by ERS (Wu et al., 2018). It was shown that ginsenoside Rg3 induced the apoptosis of gallbladder cancer cells via the ERS pathway.

## 15 Natural products target ERS-mediated apoptosis in prostate cancer

Matrine at a concentration of 4 mM inhibited proteasomal CT-like activity in prostate cancer DU145 and PC-3 cells and suppressed EMT by enhancing E-cadherin and lowering Vimentin and N-cadherin expression; moreover, matrine activated the ubiquitin‒proteasome system, promoted the UPR, released Bip, activated the ERS-mediated PERK pathway, phosphorylated eIF2α, upregulated ATF4 and CHOP expression, and triggered apoptosis by suppressing Bcl-2 and upregulating Bax; *in vivo*, similar results were achieved in DU145 xenograft mice treated with 50 mg/kg matrine, with inhibition of tumor proliferation and Ki-67 expression in tumor-bearing nude mice (Chang et al., 2018). Astragaloside (100 nmol/L) significantly upregulated the protein expression of the ERS factor BiP in prostate cancer DU145 cells and promoted ERS-mediated protein expression of IRE1, p-PERK and AFT6 without affecting the total level of PERK, thereby activating AFT4, CHOP and caspase-12-related apoptotic pathways and inducing apoptosis in prostate cancer cells (Tan et al., 2018). Excessive ROS production disrupted the pro-oxidant/antioxidant balance by exceeding the number of available intracellular antioxidants, which triggered mitochondrial dysfunction and the pro-apoptotic ERS signaling pathway, leading to apoptosis; isobavachalcone significantly decreased pro-caspase-3 levels, enhanced cleaved caspase-3 levels and activated the caspase apoptotic pathway, while isobavachalcone upregulated the expression of ERS markers XBP-1, ATF4, GRP78, Chop and p-eIF2α proteins and inhibited the enzymatic activity of the antioxidant enzyme thioredoxin reductase 1 (TrxR1) protein in a concentration-dependent manner; silencing of TrxR1 elevated the level of isobavachalcone-induced ROS, generated oxidative stress, induced lethal ERS in prostate cancer PC-3 cells and promoted apoptosis (Li et al., 2018). Quercetin lowered the protein levels of CDK2 and cyclins E and D and induced G0/G1 phase block, reduced Bcl-2 protein levels and MMP, generated Ca^2+^ and released Cyt C, upregulated Bax and caspase-3/8/9, facilitated the transportation of AIF proteins from mitochondria to the nucleus and upregulated the protein expression of ATF, GRP78 and GADD153; these data suggested that quercetin might stimulate apoptosis utilizing the mitochondrial pathway and direct activation of the caspase cascade by ERS (Liu et al., 2014). In summary, the mechanisms by which matrine, astragaloside, isobavachalcone, and quercetin induce apoptosis in prostate cancer cells are mainly the PERK and IRE1 pathways, with DU145 and PC-3 cells being the primary cell lines studied.

## 16 Natural products target ERS-mediated apoptosis in cervical cancer

Tanshinone IIA significantly enhanced the degradation of PARP, caspase-3/9, upregulated the Bax/Bcl-2 ratio, released Cyt C into the cytoplasm, upregulated the phosphorylation of p38 and JNK signaling, and hindered the cell cycle in the G2/M period in advanced cervical cancer CaSki cells, resulting in a potent growth suppression and pro-apoptotic effect on CaSki cells, suggesting that 7.5 µM tanshinone IIA induced mitochondria-associated apoptosis by stimulating the JNK and p38/Bax/caspase signaling pathways; furthermore, tanshinone IIA increased the levels of PERK, IRE1, and phosphorylated eIF2α, ATF4 and CHOP, leading to PARP degradation, and activated the p38 and ASK1 signaling pathway to promote cell death, suggesting that tanshinone IIA might induce apoptosis, at least partially, through the ERS pathway via eIF2α phosphorylation and IRE1-ASK1 activation (Pan et al., 2013). Saikosaponin-A at 15 μM notably upregulated the expression of GRP78, CHOP and caspase12 in HeLa cells, induced activation of the ERS pathway, and thus promoted apoptosis; moreover, Saikosaponin-A enhanced Bax expression, downregulated Bcl-2 expression, stimulated the expression of caspase-3, an important mediator of apoptosis, and suppressed the PI3K/AKT signaling pathway to induce apoptosis; these findings with HeLa nude mice as a model were also confirmed *in vivo* (Du et al., 2021). In cervical cancer HeLa cells, celastrol induced G2/M cell cycle arrest and caused apoptosis, parapoptosis and autophagy via various pathways, including proteasome, ERS and Hsp90 inhibition; apoptosis and parapoptosis promoted cell death, while autophagy was a cytoprotective mechanism in which endoplasmic reticulum apoptosis was caused by celastrol treatment, resulting in the significant upregulation of ERS markers Bip, PERK and IRE1 and causing cytoplasmic vacuolization that was associated with endoplasmic reticulum expansion rather than autophagy (Wang et al., 2012). In addition, 45 μM 20(S)-ginsenoside Rh2 elevated caspase 3 activity and phosphatidylserine (PS) ectopia to inhibit cervical cancer HeLa cell proliferation and promote apoptosis, and 20(S)-ginsenoside Rh2 enhanced ATF4, DDIT3, JUN, FOS, Bcl- 2-binding component 3 (BBC3) and phorbol-12-myristate-13-acetate-induced protein 1 (PMAIP1) gene expression and significantly suppressed the heterodimer of transcript DDIT3 for induction of ERS-related apoptosis in HeLa cells (Liu et al., 2022). In summary, the mechanisms by which tanshinone IIA, saikosaponin-A, celastrol, and 20(S)-ginsenoside Rh2 induce apoptosis in cervical cancer cells are predominantly the PERK and IRE1 pathways, with HeLa cells being the primary cell line studied.

## 17 Natural products target ERS-mediated apoptosis in ovarian cancer

Myricetin suppressed the viability of ovarian cancer SKOV3 cells, induced nuclear fragmentation, enhanced caspase 3 activity levels and thus induced apoptosis in SKOV3 cells; in addition, myricetin also upregulated the ERS-related proteins GRP78 and CHOP in SKOV3 cells and boosted the phosphorylation of the DNA double-strand break (DNA DSB) marker H2AX, indicating that myricetin induced DNA DSBs and ERS, promoting apoptosis in SKOV3 cells (Xu et al., 2016). It was evident that myricetin promoted apoptosis in ovarian cancer cells by stimulating ERS.

## 18 Natural products target ERS-mediated apoptosis in leukemia

In the leukemia cell lines U937 and HL-60 and primary leukemia cells (AML), asperuloside stimulated the cleavage of caspase-9/3 and PARP and facilitated the loss of MMP and the release of Cyto-c from mitochondria, inducing cell death; in addition, in U937 cells and heterozygous nude mice, asperuloside significantly induced ERS by increasing the expression levels of GRP78, PERK, eIF2α, CHOP, p-IRE1, XBP1, ATF6 and cleaved caspase-12, further showing that asperuloside regulated the interaction between GRP78 and PERK and subsequently mediated apoptosis (Rong et al., 2020). Shikonin increased ROS accumulation in Adult T cell leukemia/lymphoma (ATLL) cells, stimulated ERS and promoted the expression of p-eIF2α, ATF4, XBP-1, p-JNK, and CHOP, which induced apoptosis; moreover, shikonin suppressed the growth of ATLL cells in xenograft mice (Boonnate et al., 2023). In summary, asperuloside and shikonin promoted apoptosis in leukemia cells by regulating the ERS pathway.

## 19 Natural products target ERS-mediated apoptosis in osteosarcoma

Polyphyllin I at a dose of 1.25 mM inhibited proteasomal CT-like activity in osteosarcoma MG-63, Saos-2 and U-2 OS cells and induced cellular ERS to active the UPR and dispose of accumulated undegraded proteins; BiP expression was upregulated and its mediated PERK-eIF2α-ATF4-GADD153 (CHOP) pathway was activated, and Polyphyllin I inhibited the activity of Bcl-2 and Bcl-xL and promoted the activity of Bax and Bak, which subsequently upregulated caspase-3 and PARP protein expression and triggered apoptosis (Chang et al., 2015). In osteosarcoma U2OS cells and hormonal mice, (3R)-5,6,7-trihydroxy-3-isopropyl-3-methylisochroman-1-one (TIM) decreased MMP, promoted Cyt C release, increased DNA fragmentation, enhanced caspase-3/9 and Bax activity and lowered Bcl-2 expression, suggesting that TIM suppressed cell growth and triggered apoptosis by the process of mitochondrial death regulation; TIM also elevated the expression of the proapoptotic protein NOXA and decreased the expression of the antiapoptotic protein Mcl-1 in the apoptotic pathway, suggesting that TIM induced intracellular mitochondria through NOXA/Mcl-1 axis apoptosis; moreover, TIM upregulated the protein expression of IRE1, ATF6 and GRP78, indicating that TIM activated the ERS apoptotic pathway (Song et al., 2019). In osteosarcoma MG-63 and U2OS cells, psoralen triggered G0/G1 cell cycle arrest by reducing the levels of cyclin A1, cyclin B1, cyclin D1 and cyclin k2 and promoted apoptosis by elevating cleaved caspase 3 and Bax protein levels and declining the levels of Bcl-2 protein; additionally, psoralen upregulated the expression of DDIT3, GADD34, ER degradation-enhancing alpha-mannosidase-like 1 (EDEM1), Growth Differentiation Factor 15 (GDF15), and S1P mRNA and enhanced the levels of CHOP, IRE1, XBP1s, GRP78, PERK, and ATF-6, indicating the activation of ERS and suppression of cell proliferation, and finally psoralen-induced apoptosis (Li and Tu, 2022). In summary, Polyphyllin I, TIM, and psoralen primarily worked on U2OS cell lines and promoted apoptosis in osteosarcoma cells by activating the PERK and IRE1 pathways.

## 20 Natural products target ERS-mediated apoptosis in melanoma

Shikonin suppressed the proliferation of human melanoma A375 cells by arresting cells at the G2/M stage, inducing apoptosis and regulating autophagy in the ROS/ERS apoptotic pathway and the ROS/p38 signaling pathway, which the specific mechanism was that shikonin increased p21 levels and decreased cyclin B1 levels, leading to G2/M phase block; Shikonin produced ROS, induced ERS and upregulated p-eIF2α, CHOP and cleaved caspase-3 expression, which significantly triggered ERS-mediated apoptosis; moreover, Shikonin initiated protective autophagy by the activation of the p38 pathway, leading to increased levels of p-p38, LC3B-II and Beclin 1, suggesting that blocking autophagy could promote apoptosis (Liu et al., 2019). Shikonin triggered apoptosis in melanoma cells via the ERS pathway.

## 21 Discussion

The effects of natural products on tumors are evident, and mainly include inhibiting tumor cell growth, enhancing cell death, regulating cell autophagy, arresting the cell cycle, and reducing drug resistance in tumor cells. Our review indicates that natural products exert their antitumor effects mainly by stimulating the ERS pathway to promote the apoptosis of tumor cells. Inhibiting cell proliferation, regulating autophagy, blocking the cell cycle and alleviating drug resistance of tumor cells do not require ERS-related processes to occur. We have summarised the literature on natural products that promote tumor cell apoptosis by targeting ERS and have drawn the following conclusions:

In terms of cancer types, the stacked plot in Figure 2 revealed that the natural products targeting the ERS apoptotic pathway were predominantly studied in liver, lung, and colorectal cancers, followed by substantial research in gastric cancer, breast cancer, nasopharyngeal cancer, glioma, cervical cancer, prostate cancer, oesophageal cancer, and osteosarcoma. Conversely, studies on other cancer types were scarce, and no studies investigated pancreatic and bladder cancer. The reasons for this absence are twofold. First, since tumors with high morbidity and mortality are more dangerous and more urgently in need of specific drugs to alleviate them, experimental studies are of greater clinical significance (Li et al., 2017; Islam et al., 2022). Second, the maturity of experimental techniques for different tumors influences the extent of research, and tumors with higher morbidity and mortality possess more cell types and mature experiments (Yuan et al., 2023b). A total of 69 natural products are reviewed in this paper. However, research on various natural products is sporadic, lacking consistency, and no single product has been tested on a large scale to target their mechanism of action, and a potent natural product has yet to be selected for standardized and quantitative experiments. In the present study, bufalin, curcumin, ginsenoside, tanshinone and artemisianin were tested more frequently but only twice or thrice. Therefore, the next step is to select a more effective natural product extract, such as bufalin or curcumin, and select more experimental cancer types, such as liver and lung cancer, for ERS apoptosis pathway-related experiments. Furthermore, it is essential to include untested cancer types such as pancreatic and bladder cancer in ERS experiments to bridge the existing knowledge gap.

**FIGURE 3:**
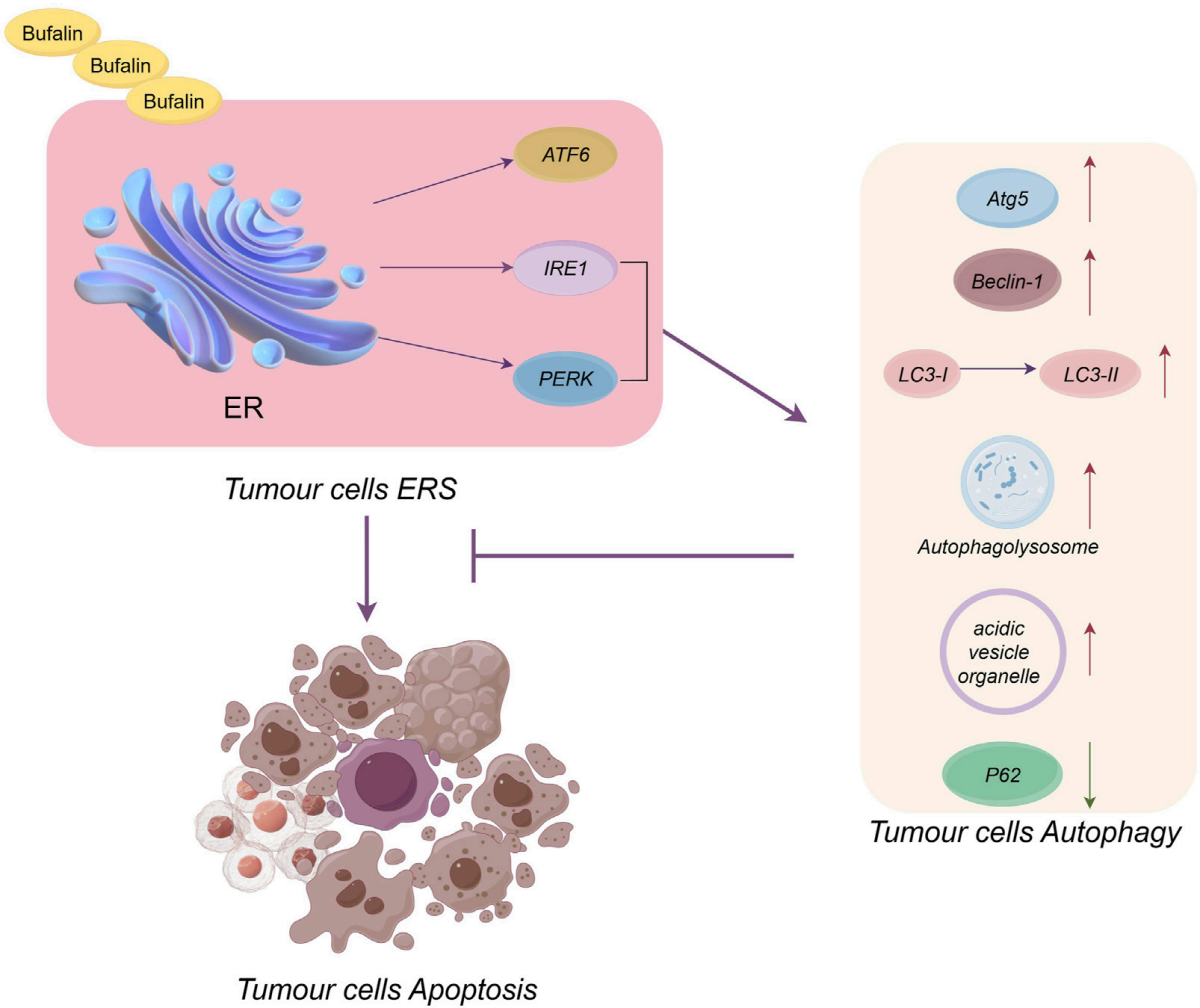
Plot of the relationship between ERS, autophagy and tumour cell apoptosis (in the case of bufalin). Bufalin promotes tumour cell apoptosis via the ERS pathway, but ERS also promotes autophagy to inhibit ERS-induced tumour cell apoptosis.

Regarding the tumor death mechanism, we found that tumor cells achieve death mainly through a combination of the mitochondrial apoptosis pathway, ERS apoptosis pathway, autophagy pathway and cell cycle inhibition pathway but through the ERS apoptosis pathway alone (Yuan M. et al., 2022; Kooshki et al., 2022; Tewari et al., 2022). This does not mean that the ERS apoptotic pathway is a concomitant pathway; on the contrary, the ERS apoptotic pathway is a key pathway for the antitumour effects of natural products, a fact that has been well demonstrated by the fact that all 69 natural products reviewed here promote tumour cell apoptosis via ERS. However, at this stage, when researchers conduct experiments on natural products, they usually measure the ERS apoptosis pathway, mitochondrial apoptosis pathway and autophagy pathway at the same time in order to have a comprehensive understanding of the anti-tumour effects of natural products. So there are fewer studies targeting the ERS apoptotic pathway alone, but this does not mean that the ERS apoptotic pathway is not critical. Given the above background, the conclusions we have drawn also confirm that the ERS apoptotic pathway, the mitochondrial apoptotic pathway and autophagy most often function simultaneously, and that natural product-induced tumour cell death is mainly achieved through these combined pathways, and that related studies have focused on these three pathways. Regarding the link between the autophagic and apoptotic pathways, a study claimed that inhibition of ERS-mediated autophagy in ATF4-associated cells could play an anticancer role (Yang et al., 2023). The results of this paper show that natural products promote ERS in order to promote apoptosis in tumour cells, and in the process ERS also mediates autophagy, which is primarily a protective pathway whose mechanism of action is to inhibit apoptosis by mitigating ERS and promoting the restoration of endoplasmic reticulum homeostasis. We explained the relationship between autophagy, ERS and tumour cell apoptosis using the most frequently occurring bufalin as an example, see Figure 3. From the figure, it can be seen that bufalin induces ERS through ATF6, PERK and IRE1 pathways, immediately followed by apoptosis of tumour cells. Whereas IRE1 and PERK pathways can induce changes in autophagy-related markers such as increased Atg5, LC3-II, *etc.*, to promote autophagy, which inhibits ERS-induced apoptosis in tumour cells. It can be seen that autophagy and ERS apoptosis are antagonistic to each other. However, there are relatively few studies on the interaction between autophagy and ERS apoptosis, and at the same time autophagy is one of the important pathways of tumour cell death. Influenced by the above reasons, the next step needs to continue the interaction study between autophagy and ERS apoptosis to determine whether autophagy only unilaterally inhibits ERS apoptosis, and whether it can promote ERS apoptosis. Therefore, it is imperative to investigate the interactions between autophagy and ERS to understand their intrinsic mechanisms, and it is crucial to study how autophagy promotes ERS-induced apoptosis. At the same time, the next step should be to conduct studies on the apoptosis-promoting pathway of ERS alone, and it is also worthwhile to explore how the combination of mitochondrial apoptosis, autophagy death and ERS apoptosis can better promote apoptosis. It can also be seen from this paper that both underexpression and overexpression of ERS can cause apoptosis. Natural products regulate apoptosis mainly by upregulating the expression of GPR78 or CHOP-related proteins. However, inhibiting ERS by methods such as reducing the expression levels of PERK, eIF2α and CHOP, has rarely been studied, with only three cases mentioned in this paper. Meanwhile for the ERS-related apoptosis pathway, most of the experimental proteins and pathways studied at this stage are two related pathways mediated by PERK and IRE1. In contrast, the ATF6 pathway, also an ERS apoptotic pathway, has rarely been studied, especially since the studies related to natural products were close to zero. Therefore, further research is needed to identify the mechanisms by which natural products inhibit ERS and promote apoptosis under inadequate ERS expression. Additionally, studies on promoting apoptosis by ATF6-related pathways and proteins under the condition of ERS overexpression are required to improve the understanding of the ERS apoptosis pathway. Finally, studies on the relationship between ERS, ROS levels and calcium are insufficient and should be conducted.

In terms of experimental techniques, it mainly rely on cell and mouse experiments (Voskoglou-Nomikos et al., 2003), as no clinical studies involving humans have yet been conducted. We believe that the lack of clinical trial evidence may be due to the following reasons: 1. Since the antitumor pathway of natural product-targeted ERS is not yet completely clear and the mechanism is still controversial, the relevant drugs have not been developed or are still in the beginning stage of research and development, and cannot be subjected to clinical trials for the time being. 2. Due to the lack of efficacy or safety issues, as well as differences in the control of clinical trials in different countries, it is not possible to ensure the smooth progress of the trials, and not every trial will achieve the expected results. 3. The threat of cancer is too great, and patients are often unable to overcome the psychological pressure to face the clinical trials, which is a great obstacle to the development of the trials and the collection of data in later stages. Therefore, a commonly used natural product targeting the ERS apoptotic pathway, such as bufalin, first needs to be selected for drug development. The next crucial step is conducting large-scale clinical studies and evidence-based medical research to identify drugs that effectively treat malignant tumors. Additionally, there have been limited safety studies on natural products, likely due to the small number of experimental studies on natural products targeting ERS (Atanasov et al., 2021). Therefore, the next step is to perform safety studies and pharmacokinetic and pharmacological analyses of natural products to ensure the safety of natural products.

In summary, natural products have the ability to modulate the ERS pathway to promote apoptosis in various cancer cells. Nevertheless, the relevant research content is insufficient, the mechanism is unelucidated, no relevant clinical studies have been performed, and safety studies are inadequate. The next step is to identify an effective natural product that targets ERS and to conduct quantitative cellular or animal experiments on one type of tumor cell from common cancer types or many different types of tumor cells from various cancer types to clarify the three ERS apoptotic pathways, focusing on the ATF6-related apoptotic pathway. Then, evidence-based medical research, clinical trials, safety testing, and pharmacokinetic and pharmacological analyses of natural products can be executed to develop novel drugs and optimize the antitumor effects of natural products.

## Figures and Tables

**Table:** 

**5-FU**	5-Fluorouracil
**ABCG2**	ATP-binding cassette, subfamily G (WHITE), member 2
**AIF**	Apoptosis Inducing Factor
**Akt**	protein kinase B
**AMPK**	AMP-activated protein kinase
**ASK1**	apoptosis signal-regulating kinase 1
**ATF4**	activating transcription Factor 4
**ATF6**	activating transcription Factor 6
**Atg5**	autophagy-related protein 5
**ATG7**	Autophagy Related Protein 7
**ATLL**	Adult T cell leukaemia/lymphoma
**Bax**	BCL2-associated X
**BBC3**	Bcl-2-binding component 3
**Bcl-2**	B-cell lymphoma-2
**BiP**	heavy-chain binding protein
**Caspase**	cysteinyl aspartate specific proteinase
**CDK**	cyclin-dependent kinases
**CDK2**	cyclin-dependent kinases 2
**CDK4**	cyclin-dependent kinases 4
**CDK6**	cyclin-dependent kinases 6
**CHAC1**	cation transport regulator-like protein 1
**CHOP**	C/EBP homologous protein
**CLIC1**	Chloride Intracellular Channel Protein 1
**Cyt C**	cytochrome c
**DAPK3**	death-associated protein kinase 3
**DDIT3**	DNA damage inducible transcript 3
**DNA DSB**	DNA double-strand break
**DR5**	death receptor 5
**E2F1**	E2F Transcription Factor 1
**EDEM1**	ER degradation-enhancing alpha-mannosidase-like 1
**EEC**	ethanolic extract of *C. cicadae*
**eIF2α**	eukaryotic translation initiation Factor 2 α
**EMT**	epithelial-mesenchymal transition
**ERK**	extracellular regulated protein kinases
**ERP57**	endoplasmic reticulum resident protein 57
**ERP72**	endoplasmic reticulum resident protein 72
**ERS**	endoplasmic reticulum stress
**FOS**	Fos proto-oncogene
**GADD34**	growth arrest and DNA damage protein 34
**GADD153**	growth arrest and DNA damage-inducible 153
**GBM**	glioblastoma multiforme; GDF15, Growth Differentiation Factor 15
**GRh2**	Ginsenoside Rh2
**GRP78**	glucose regulated protein 78
**GRP94**	glucose regulated protein 94
**HCC**	hepatocellular carcinoma cells
**IC**_**50**_	half maximal inhibitory concentration
**IRE1**	inositol-requiring enzyme 1
**IRE1α**	inositol-requiring enzyme 1 α
**JNK**	c-Jun N-terminal kinase
**KGM**	Konjac glucomannan
**MAPK**	mitogen-activated protein kinase
**Mcl-1**	myeloid cell leukemia-1
**MMP**	Mitochondrial membrane potential
**mTOR**	mammalian target of rapamycin
**NF-κB**	nuclear factor kappa-B
**NLRP3**	NOD-like receptor pyrindomain-containing protein3
**NSCLC**	non-small cell lung cancer
**PARP**	poly ADP-ribose polymerase
**PDI**	protein disulfide isomerase
**PERK**	protein kinase R (PKR)-like endoplasmic reticulum kinase
**PI3K**	phosphatidylinositol 3-kinase
**PMAIP**	phorbol-12-myristate-13-acetate-induced protein 1
**ROS**	reactive oxygen species
**SERCA2**	Ca^2+^-ATPase 2
**TCF4**	Transcription factor 4
**TLR4**	Toll-like receptor 4
**TNF**	tumor necrosis factor
**TP53**	Tumor protein p53
**TRAF2**	TNF receptor-associated Factor 2
**TRIB3**	Tribbles pseudokinase 3
**TrxR1**	thioredoxin reductase 1
**TTF1-NPs**	5,2′,4′-trihydroxy-6,7,5′-trimethoxyflavone nanoparticles
**UPR**	unfolded protein response
**VEGF**	vascular endothelial growth factor
**VSOR**	volume-sensitive outwardly rectifying
**XBP1**	X-box binding protein 1
